# Intramacrophage RIL-seq uncovers an RNA antagonist of the *Salmonella* virulence-associated small RNA PinT

**DOI:** 10.1093/nar/gkaf1364

**Published:** 2025-12-22

**Authors:** Hoda Kooshapour, Gianluca Matera, Elisa Venturini, Leona Metka, Thorsten Bischler, Jörg Vogel, Alexander J Westermann

**Affiliations:** Department of Microbiology, Biocenter, University of Würzburg, Würzburg D-97074, Germany; Helmholtz Institute for RNA-based Infection Research (HIRI), Helmholtz Centre for Infection Research (HZI), Würzburg D-97080, Germany; Helmholtz Institute for RNA-based Infection Research (HIRI), Helmholtz Centre for Infection Research (HZI), Würzburg D-97080, Germany; Helmholtz Institute for RNA-based Infection Research (HIRI), Helmholtz Centre for Infection Research (HZI), Würzburg D-97080, Germany; Department of Microbiology, Biocenter, University of Würzburg, Würzburg D-97074, Germany; Helmholtz Institute for RNA-based Infection Research (HIRI), Helmholtz Centre for Infection Research (HZI), Würzburg D-97080, Germany; Core Unit Systems Medicine, University of Würzburg, Würzburg D-97080, Germany; Helmholtz Institute for RNA-based Infection Research (HIRI), Helmholtz Centre for Infection Research (HZI), Würzburg D-97080, Germany; Institute of Molecular Infection Biology (IMIB), University of Würzburg, Würzburg D-97080, Germany; Department of Microbiology, Biocenter, University of Würzburg, Würzburg D-97074, Germany; Helmholtz Institute for RNA-based Infection Research (HIRI), Helmholtz Centre for Infection Research (HZI), Würzburg D-97080, Germany

## Abstract

*Salmonella* virulence chiefly relies upon two major pathogenicity islands, SPI-1 and SPI-2, which enable host cell invasion and intracellular survival, respectively. Growing evidence suggests post-transcriptional control of SPI gene expression by Hfq-dependent small regulatory RNAs (sRNAs) such as PinT. This 80-nucleotide sRNA is highly expressed after *Salmonella* enters host cells and modulates the transition from the SPI-1 to SPI-2 program by targeting the mRNAs of different virulence factors. However, it remains unclear how PinT activity can be counteracted when the suppression of virulence genes needs to be relieved. Here, we mapped the RNA interactome of *Salmonella* recovered from infected macrophages, using an optimized version of RIL-seq. In addition to offering an unprecedented view of Hfq-mediated RNA interactions during *Salmonella*’s intracellular infection stage, RIL-seq uncovered the previously described 3′ end-derived sRNA InvS as a direct negative regulator of PinT. Biochemical and genetic experiments suggest a decoy mechanism by which InvS lifts PinT-mediated target repression. Moreover, InvS acts as an mRNA repressor of the adhesion protein MipA and PinT interaction with InvS relieves *mipA* repression. Together, our work identifies a pair of antagonistic sRNAs in a growing post-transcriptional network of virulence gene regulation.

## Graphical Abstract

**Graphical Abstract:**
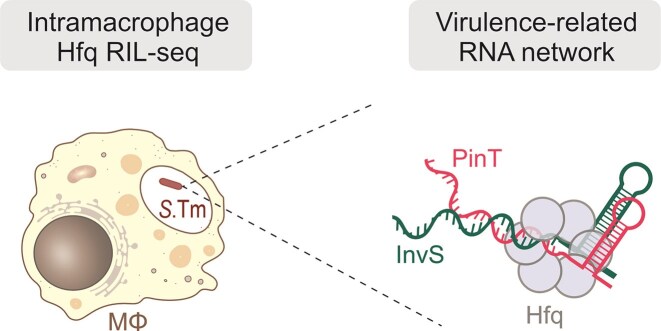

## Introduction

*Salmonella enterica* represents an important species of zoonotic and foodborne pathogens, responsible for a range of diseases in livestock and humans, from self-limiting gastroenteritis to potentially life-threatening typhoid fever in immunocompromised patients [1]. Being a facultative intracellular pathogen, *Salmonella* can establish a replication niche inside epithelial and phagocytic host cells in the gut. *Salmonella*’s ability to infect mammals is mediated by intricate genetic systems, including the well-characterized *Salmonella* Pathogenicity Islands (SPIs) 1 and 2. These islands each encode an arsenal of secreted virulence effector proteins and the necessary type-III secretion systems (T3SS-1 and -2) to translocate these effectors into eukaryotic host cells [2, 3]. *Salmonella* infection is driven by sequential waves of expression of SPI-1 and SPI-2 genes, which mediate bacterial invasion and replication inside host cells, respectively [4, 5]. Numerous transcriptional regulators have been identified, which collectively ensure that SPI gene expression—which is costly and requires coordination with other cellular pathways—is only activated under infection-conducive conditions. There is also growing evidence for a crucial role of post-transcriptional control of SPI gene expression, following observations that genetic inactivation of the global RNA-binding proteins CsrA, Hfq, and ProQ resulted in attenuated virulence [6–8]. Among these, Hfq is a conserved RNA-binding protein that forms a homohexameric ring of ∼60 kDa, which primarily acts to mediate short base-pairing interactions of associated small regulatory RNAs (sRNAs) with their target messenger RNAs (mRNAs) [9–12]. *Salmonella* species encode ∼100 Hfq-associated sRNAs with diverse functions in bacterial physiology [13–17], including validated or predicted regulations of virulence factors via mRNA targeting [14, 18–25].

The most extensively characterized virulence-related sRNA in *Salmonella* species to date is PinT. Originally described as candidate STnc440 by sequencing Hfq-associated transcripts in *Salmonella enterica* serovar Typhimurium [13], PinT is an 80 nt-long sRNA from a horizontally acquired *Salmonella*-specific locus that also encodes RtsA, a co-activator protein of invasion genes [18]. PinT expression is positively regulated by PhoP/Q [18], the SPI-2-activating two-component system that is essential for intracellular survival [26, 27]. Moreover, dual RNA-seq profiling of different cell types has shown PinT to be the top-induced sRNA as *Salmonella* invades and replicates in host epithelial cells and macrophages [18]. Through repression of multiple mRNAs of specific SPI-1 and SPI-2 regulators and effectors, PinT influences the host response by modulating the inflammatory signaling pathways triggered during infection [18]. A comprehensive transposon insertion sequencing screen linked *pinT* disruption to significant fitness defects in pigs and cattle, highlighting its role in host colonization [28]. Over the past years, the PinT regulon has continuously been expanded. It now includes seven direct target mRNAs, with mostly virulence-related functions [18, 20, 21]. The previous work on PinT has established it as an exemplary sRNA that controls the temporal expression of two major virulence programs, thus facilitating the pathogen’s transition from host cell invasion to ensuring intracellular replication [18, 29]. However, sustained repression of virulence and metabolic regulators by PinT would interfere with the bacterium’s ability to survive intracellularly. Thus, a crucial unanswered question is how the activity of this sRNA is counteracted to eventually relieve PinT-mediated target repression during later infection stages.

RNA interaction by ligation and sequencing (RIL-seq) allows to chart the complex network of Hfq-associated RNA–RNA interactions within bacterial cells in a single experiment [30]. The method involves the ligation of physically interacting RNA pairs, followed by their co-purification with Hfq and high-throughput sequencing of the resulting chimeric fragments [30–33]. Amongst other findings, RIL-seq and related techniques have substantially contributed to our emergent appreciation of the prevalence of so-called “sRNA sponges,” that is RNA molecules that can sequester sRNAs, preventing them from regulating their respective target mRNAs [34]. As a recent example, application of RIL-seq to *Salmonella* grown in a SPI-1-inducing condition led to the discovery of OppX, an RNA sponge that acts antagonistically to the Hfq-associated sRNA MicF, which is a porin repressor [35]. Of note, however, the global Hfq-associated RNA-RNA interactome has not previously been charted during the intracellular stage of this—or any other—pathogen’s infection cycle, despite the relevance of the intracellular *Salmonella* subpopulation for disease development [36].

To advance the characterization of virulence-related sRNAs , the current work pioneers the application of RIL-seq to bacteria growing inside eukaryotic cells. By systematically determining the minimal input material compatible with the RIL-seq protocol, we were able to capture the Hfq-associated RNA interactome of intramacrophage *Salmonella*. In addition to confirming previously predicted target mRNAs of PinT, our RIL-seq results identified another horizontally acquired Hfq-associated sRNA, InvS, as a key interactor of PinT. Follow-up characterization showed that InvS acts to sequester PinT, thereby relieving the repression of PinT target mRNAs. Moreover, our RIL-seq data indicated that the *mipA* (aka *ompV*) mRNA, which encodes an outer membrane protein involved in bacterial adhesion, is the primary target of InvS. Collectively, this study reveals a complex interplay of two Hfq-associated sRNAs and their targets in timing *Salmonella* virulence gene expression and adds virulence to the emergent theme of sRNA regulation by RNA decoys and sponges [37, 38].

## Materials and methods

### Bacterial strains and growth conditions

*Salmonella enterica* serovar Typhimurium strain SL1344 (JVS-1574) was used as a wild-type strain. The complete list of strains used in this study is contained in Supplementary Table S2. Bacterial cells were grown at 37°C with shaking at 220 rpm in LB medium. Where appropriate, media were supplemented with antibiotics at the following concentrations: 100 µg/ml ampicillin, 50 µg/ml kanamycin, and 20 µg/ml chloramphenicol. To investigate bacterial behavior under SPI-1-inducing conditions, cultures were grown in LB until an OD_600_ of 2.0 was reached. For simulating SPI-2-inducing conditions, cultures were first cultivated in LB to an OD_600_ of 2.0, followed by two successive washes with phosphate-buffered saline (PBS). Subsequently, the bacteria were inoculated at a ratio of 1:50 and transferred to SPI-2 medium (170 mM MES, 5 mM KCl, 7.5 mM (NH_4_)_2_SO_4_, 0.5 mM K_2_SO_4_, 1 mM KH_2_PO_4_, 8 µM MgCl_2_, 38 mM glycerol, and 0.1% bacto casamino acid, pH 5.8). This cultivation was conducted at 37°C with constant shaking at 220 rpm until the cultures reached an OD_600_ of 0.3.

### Western blot analysis

Western blots were performed following previously published procedures. In brief, bacterial cultures were collected by centrifugation for 4 min at 13 000 *× g* at 4°C, and the pelleted cells were dissolved in 1× protein loading dye to a final concentration of 0.01 OD/µl. The samples were heated up for 10–15 min at 95°C, and 0.1 ODs were separated on a 12.5% polyacrylamide gel. Proteins were transferred onto a nitrocellulose membrane for 90 min at 0.34 A, using a semi-dry blotter in transfer buffer (25 mM Tris–HCl pH 8.3, 190 mM glycine, and 20% methanol). Membranes were blocked with 5%–10% milk for 1 h at room temperature and rinsed in 1× TBS-Tween buffer (20 mM Tris–HCl pH 7.6, 150 mM NaCl, and 0.1% Tween20). After blocking, membranes were incubated with the primary antibodies [monoclonal α-FLAG, Sigma–Aldrich #F1804; polyclonal α-GroEL, Sigma–Aldrich #G6532; custom-made, rabbit, polyclonal α-MipA, Eurogentec (Supplementary Table S2)] diluted in 1× TBS-Tween buffer containing 3% BSA. The membranes were washed three times for 15 min with agitation in 1× TBS-Tween buffer. Membranes were then incubated for 1 h at room temperature with HRP-linked secondary antibodies [α-mouse or α-rabbit, Cell Signaling Technology #7076 or #7074 (Supplementary Table S2)], diluted in 1× TBS-Tween containing 3% BSA, and washed three times for 15 min with 1× TBS-Tween. The membranes were developed using Amersham ECL Prime reagents (GE Healthcare) and signals were detected on a LAS4000 and Imager 600 (GE Healthcare). Bands were quantified using ImageJ software.

### *Salmonella* infection assay

T75 cell flasks were seeded with 200 000 murine RAW264.7 macrophage cells at passage #16. On the day of the infection, macrophage cells were counted and overnight bacterial cultures were harvested to an amount that would determine a multiplicity of infection (MOI) of 50 bacteria per macrophage for all the needed flasks (# bacterial cells = [MOI × # of eukaryotic cells × # of flasks]/bacterial concentration). Before infection, harvested bacteria were opsonized with 10% of mouse serum for 20 min at room temperature. Opsonized bacteria were resuspended in RPMI medium to the correct dilution for the envisaged MOI and inoculated in cell-containing flasks. Flasks were centrifuged at 250 *× g* for 10 min at room temperature to enhance bacterial contact with host cells and synchronize the infection. Flasks were incubated for 30 min at 37°C for phagocytosis to take place. The RPMI medium from the flasks was replaced with fresh RPMI containing 100 μg/ml of gentamicin (high-gentamicin) to inhibit extracellular bacteria and flasks were incubated at 37°C for 30 min. RPMI medium was replaced once more with RPMI containing 10 μg/ml of gentamicin (low-gentamicin) and incubated for the remainder of the assay. At 20 h post-infection (p.i.), flasks were washed once with ice-cold 1× PBS and incubated with 10 ml of 0.1% PBS-Triton X-100 (PBS-T) to detach adhering cells. Harvested cells were either directly subjected to RIL-seq (strategy 1) or incubated for 10 min at room temperature in 0.1% PBS-T to ensure mild lysis of only the eukaryotic cells (strategy 2). In case of the latter, samples were then centrifuged at 250 × *g* for 10 min at 4°C to separate eukaryotic cell debris from formerly intracellular bacteria and the bacteria-containing supernatant was collected and centrifuged at 4500 × *g* for 20 min at 4°C. The resulting bacterial pellet was snap-frozen in liquid nitrogen.

For competitive infection assays between different *Salmonella* strains, we mixed equal bacterial numbers of each strain following opsonization, as described [39]. These mixtures were used for infection of RAW264.7 cells at a total MOI of 25. At 20 h p.i., the competitive fitness of the strains was deduced from strain-specific colony-forming units (CFUs) upon plating serial dilutions of the lysates on LB agar, thanks to strain-specific fluorescent markers (sfGFP and mScarlet) detected using a blue light transilluminator.

### RIL-seq

RIL-seq experiments were performed as described in [30, 40] with a few modifications to adapt the protocol to *Salmonella*. Briefly, *Salmonella* strains carrying a wild-type or a 3×FLAG-tagged copy of *hfq* were grown in LB medium to an OD_600_ of 2.0. The samples were cross-linked under a 256 nm UV light source and pelleted in ice-cold 1× PBS. Pellets were lysed in NP-T buffer (50 mM NaH_2_PO_4_, 300 mM NaCl, and 0.05% Tween, pH 8.0) supplemented with protease inhibitor (1:200) and RNase inhibitor (final concentration of 0.1 U/µl). Lysates were incubated with anti-FLAG antibody-bound protein A/G magnetic beads for 2 h at 4°C under continuous rotation, followed by three washing steps with lysis buffer. Beads were treated with an RNase A/T1 mix for 5 min at 22°C in an RNase inhibitor-free lysis buffer. Samples were washed three times with lysis buffer supplemented with 3.25 µl of SuperaseIN. The trimmed ends of RNAs were cured by PNK treatment for 2 h at 22°C with agitation, followed by two washing steps at 4°C. Proximal Hfq-bound RNAs were ligated with T4 RNA ligase I in the following reaction: 8 µl of T4 ligase buffer, 7.2 µl of dimethyl sulfoxide (DMSO), 0.8 µl of ATP (100 mM), 32 µl of PEG 8000, 1.2 µl of RNase inhibitor, 23.6 µl of water, and 140 U of T4 RNA ligase I. Samples were incubated overnight at 22°C with agitation, followed by three steps of washing with lysis buffer at 4°C. The RNAs were eluted from beads with a proteinase K digestion for 2 h at 55°C followed by LS Trizol extraction, as per manufacturer instructions. Purified RNA was resuspended in 7 µl of nuclease-free water and quality-controlled on a Bioanalyzer Pico RNA chip prior to cDNA library preparation.

Library preparation was conducted using the sRNA NEBNext kit for Illumina, with few modifications. Briefly, 3 µl of RNA were mixed with 1 µl of 3′ SR Adaptor (1:10 diluted) and incubated at 70°C for 2 min. 6.5 µl of 3′ ligation mix (5 µl of 3′ ligation buffer and 1.5 µl of 3′ enzyme mix) were added to each tube and incubated at 25°C for 1 h. 2.75 µl of SR mix (2.5 µl of water and 0.25 µl of SR RT primer) was added. The reactions were incubated in three sequential steps (75°C for 5 min, 37°C for 15 min, and 25°C for 15 min). Pre-denatured 5′ adaptor (1:10 diluted) was added to each tube together with 5′ ligation mix (0.5 µl of 5′ ligation reaction buffer and 1.25 µl of 5′ ligation enzyme mix), and samples were incubated at 25°C for 1 h. First-strand cDNA synthesis was initiated by adding 5 µl of cDNA mix (4 µl of first-strand buffer, 0.5 µl of murine RNase inhibitor, and 0.5 µl of SuperScript II RT). The reactions were incubated at 50°C for 1 h followed by 15 min at 70°C. 10 µl of cDNA were polymerase chain reaction (PCR)-amplified with barcoded NEB index primer and SR primer in a 50 µl of reaction (25 µl of LongAmp Taq 2× mix, 12.5 µl of nuclease-free water, 1.25 µl of SR primer, and 1.25 µl of index primer). The PCR cycling program was set as follows: 30 s at 94°C initial denaturation, 15 s at 94°C, 30 s at 62°C, 70°C for 18–20 cycles, and a final elongation for 5 min at 70°C. PCR products were purified using AMPure XL beads and checked on a DNA Bioanalyzer to estimate the size distribution and amount of DNA fragments. Amplified cDNAs from different samples were pooled in equimolar ratio and sequenced in paired-end mode with 2 × 40 or 2 × 38 cycles on an Illumina NextSeq 500 platform. All sequencing data were uploaded on the Gene Expression Omnibus (GEO) platform under the accession number GSE293893.

Raw read pairs were quality and adapter trimmed via Cutadapt [41] v2.5 in paired-end mode using a cutoff Phred score of 20. Read pairs without any remaining bases in at least one read of a pair were discarded (parameters: –nextseq-trim = 20 -m 1 -a AGATCGGAAGAGCACACGTCTGAACTCCAGTCAC -A GATCGTCGGACTGTAGAACTCTGAACGTGTAGATCTCGGTGGTCGCCGTATCATT). Processed read pairs were further analyzed using the RIL-seq software package [30, 40] v0.82 (https://github.com/asafpr/RILseq; see https://github.com/yairra/RILseq for latest versions) installed via Bioconda [42]. Here, we applied the *ad hoc* annotation of *Salmonella* Typhimurium strain SL1344 described in [15]. Briefly, gene annotations from NCBI were used for the genomic features such as transfer RNAs (tRNAs), ribosomal RNAs (rRNAs), and coding sequences (CDSs). Transcriptional units were defined based on the TSS annotation of [2] and Rho-independent terminator predictions using RNIE [43]. The BioCyc annotation [44] was used for the plasmids. In the first step, read pairs were aligned to the *Salmonella enterica* subsp. *enterica* serovar Typhimurium str. SL1344 reference genome (RefSeq assembly accession: GCF_000210855.2) using the map_single_fragments.py script with default parameters. BWA [45] v0.7.17 was used for read alignment with the RIL-seq software. A transcript file (TF) with transcriptional unit annotations was generated and included for chimera mapping based on the same reference genome as above by applying the map_chimeric_fragments.py script separately to each of the previously generated BAM files. For this, default parameters were used except for setting the parameter -t TF to incorporate the transcript file in the analysis. For subsequent analyses, chimeras from conditions with two replicates were unified as described in [30]. Finally, overrepresented interacting regions were identified using the RILseq_significant_regions.py script with default parameters except for including a BioCyc data folder for *Salmonella* (–bc_dir), the chromosome ID mapping –BC_chrlist NC_016810.1, NC_016 810,NC_017718.1, NC_017 718, NC_017719.1, NC_017 719, NC_017720.1, and NC_017 720) and parameters for excluding rRNA interactions (–ribozero –rrna_list rRNA and RRNA). The resulting significant interacting regions (S-chimeras) were reannotated using the *Salmonella ad hoc* annotation described above. In contrast to chromosomal genomic features (5′UTR, CDS, 3′UTR, sRNA, and tRNA), plasmid-derived transcripts, which almost exclusively consist of CDS regions, were assigned to feature type “exon” in the analysis. This transcript class was renamed to “Plasmid” for clarification. Summary statistics for sequencing and mapping were calculated as suggested in [30] and can be found in Supplementary Table S1. A filter of ≥40 was applied to the number of chimeric fragments supporting the S-chimeras. From the resulting, filtered dataset, RIL-seq-related figures were generated as previously described [35]. BED files for visualization of the InvS-*mipA* interaction based on aligned chimeric fragments in the IGB genome browser were generated using the generate_BED_file_of_endpoints.py script from the RIL-seq software.

**Figure 1.**
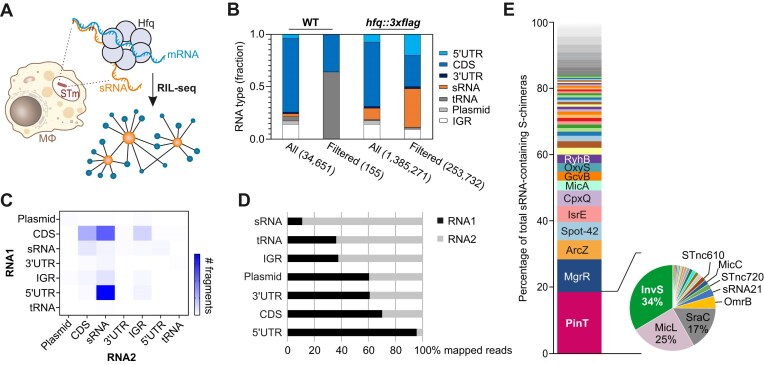
Intramacrophage RIL-seq workflow and global results. (**A**) Cartoon representation of the intramacrophage RIL-seq procedure, from *Salmonella* inside RAW264.7 macrophages to the Hfq-associated RNA network depiction. (**B**) Representation of the distribution of RNA types for significant chimeric fragments (number of fragments in S-chimeras; unfiltered [all] or filtered for ≥ 40 interactions) within the RIL-seq dataset derived from the wild-type and *hfq::3×FLAG* strains. UTR, untranslated region; CDS, coding sequence; sRNA, small regulatory RNA; tRNA, transfer RNA; plasmid, plasmid-encoded transcript; IGR, intergenic region. (**C**) Two-dimensional heat map plotting the number of chimeric fragments corresponding to abundance-filtered S-chimeras according to the position of the RNA within the chimera (RNA1 = first read, RNA2 = second read). (**D**) Distribution of chimeric fragments in RNA1 versus RNA2 for each RNA type. (**E**) The stacked bar chart denotes the relative abundance of sRNAs in S-chimeras. The pie chart to the right represents the total number of PinT interactions with various noncoding RNAs. The data plotted in panels (B–E) refer to unified values across two biological replicate intramacrophage RIL-seq experiments.

The RIL-seq dataset is accessible through an online RNA–RNA interactome browser (https://resources.helmholtz-hiri.de/rilseqset2025/) that was set up using an existing Python-based framework for visualization of RIL-seq data [35] allowing users to explore the identified post-transcriptional networks.

### Translational GFP reporter assays

Strains carrying a superfolder-GFP reporter plasmid [46] were grown as described above and streaked on LB-agar followed by UV exposure to visualize GFP expression. For the fluorescence reporter assay, specific strains were grown overnight in LB or SPI-2 media and the next day, subcultures with a starting OD_600_ of 0.02 were transferred to a 96-well plate (BRAND, #781971). GFP signal intensities and cell densities were measured every 10 min for 24 h, while the plate was continuously shaking (200 rpm) at 37°C in a BioTek Synergy H1 Plate Reader. Wells containing pure medium were included and their background fluorescence and opacity used as blanks.

### *In vitro* transcription and RNA labeling

200 ng of a DNA fragment PCR-amplified from *Salmonella* genomic DNA was used as a template in a T7 transcription reaction using the MEGAscript T7 Transcription kit (Thermo Fisher Scientific). The size and integrity of the resulting RNA product was confirmed on a denaturing polyacrylamide gel. RNA bands were excised from the gel and eluted in RNA elution buffer (0.1 M sodium acetate, 0.1% SDS, and 10 mM EDTA at 4°C overnight), isolated with Phenol:Chloroform:Isoamyl (P:C:I) and precipitated in EtOH. 50 pmol of RNA was dephosphorylated with 10 units of calf intestinal phosphatase (CIP, New England Biolabs) in a 50 μl reaction at 37°C for 1 h. CIP-treated RNA was extracted with P:C:I and EtOH precipitated. 20 pmol of the dephosphorylated RNA was 5′-labeled with 2 μl of ^32^P-γ-ATP (10 μCi/μl) using 1 unit of T4 polynucleotide kinase (Thermo Fisher Scientific) for 1 h at 37°C in a 20 μl reaction. RNA was purified from unincorporated nucleotides with microspin G-50 columns (GE Healthcare) according to the manufacturer’s instructions.

### Electrophoretic mobility shift assay

0.04 pmol of radio-labeled RNA was used for each reaction mix. Labeled RNA was denatured at 95°C for 1 min and chilled on ice for 5 min. 1× structure buffer (10 mM Tris–HCl pH 7.0, 0.1 M KCl, 10 mM MgCl_2_) was added and the RNA was re-natured at 37°C for 10 min. Per reaction, 1 μg of yeast RNA was added to the mix, and the labeled RNA was added to tubes containing increasing concentration of unlabeled RNA. Where applicable, 100 nM of purified Hfq were added to the reaction mix. Reactions were incubated at 37°C for 15 min, stopped by adding 5× RNA native loading buffer, and separated on native 6% polyacrylamide gels at 4°C in 0.5% TBE at constant current of 40 mA for 3–4 h. Gels were dried and signals detected on a Typhoon FLA 7000 phosphoimager and quantified with ImageJ software.

### RNA structure probing

Labeled RNAs were prepared as described for the EMSA. For the reactions, 0.4 pmol of labeled RNA were denatured as described earlier and incubated with increasing concentration of unlabeled RNA partner (InvS) for 15 min at 37°C in the presence of 1× structure buffer and 1 µg of yeast RNA in 10 µl. The reactions were treated with 2 µl of 25 nM lead-acetate and incubated for 90 s at 37°C. 12 µl of GL II RNA loading dye were added to each tube to stop the reaction. 10 µl of each sample were boiled at 95°C for 3 min, loaded on a 10% PAA 7 M urea gel, and separated for 3 h at 45 W. For the control lane, 1 pmol of labeled RNA was denatured at 95°C in 10 µl of water and stopped on ice with 10 µl of GL II RNA loading dye. For the OH ladder, 1 pmol of labeled RNA was denatured at 95°C for 5 min in 1× alkaline buffer in a 10 µl reaction. For the T1 ladder, 1 pmol of RNA was denatured in water for 1 min at 95°C followed by addition of RNase T1 enzyme and incubated for 3 min at 37°C. All reactions were stopped as mentioned before.

### Northern blot analysis

Four OD equivalents of bacterial cultures were snap-frozen in liquid nitrogen after adding 0.2% v/v of stop solution (95% ethanol and 5% phenol). Bacterial cells were pelleted and lysed by adding 600 µl of lysozyme (0.5 mg/ml) and 60 µl of 10% SDS, followed by a 2-min incubation at 64°C. Subsequently, 66 µl of 3 M sodium acetate (pH 5.2) and 750 µl of phenol were added, with the mixture incubated for 6 min at 64°C. After centrifugation, the aqueous phase was transferred to a PLG tube, followed by the addition of 750 µl of chloroform. After a second centrifugation, the aqueous phase was collected. RNA was then precipitated overnight at −20°C using a 30:1 mixture of ethanol and 3 M sodium acetate (pH 6.5), pelleted, washed with 75% ethanol, and resuspended in 50 µl of nuclease-free water. 5 to 10 μg of total RNA were denatured at 95°C for 5 min in RNA loading dye (95% v/v formamide, 10 mM EDTA, 0.1% w/v xylene cyanole, and 0.1% w/v bromophenol blue) and separated by gel electrophoresis in a 6% polyacrylamide/7 M urea gel in 1× TBE buffer for 2 h at 300 V. RNA was transferred onto a Hybond-XL nylon membrane (GE Healthcare) with electro-blotting at 50 V for 1 h at 4°C. The membrane was cross-linked at 120 mJ/cm^2^ with UV light and pre-hybridized for 10 min in Rapid-Hyb buffer (Amersham). A [^32^P]-labeled oligonucleotide probe was added onto the membrane and hybridized at 42°C overnight with rotation. The membrane was washed three times for 15 min with 5× SSC/0.1% SDS (first wash), 1× SSC/0.1% SDS (second wash), and 0.5× SSC/0.1% SDS (third wash) buffers at 42°C. Air dried membranes were then exposed onto a phosphor screen and signals were visualized on a Typhoon scanner and quantified with ImageJ software.

### RNA stability assay

Cultures in SPI-2 medium with an optical density of 0.3 were treated with rifampicin at a final concentration of 500 μg/ml. At specified time points post-rifampicin treatment, samples were taken for total RNA isolation. The extracted RNA samples were subsequently subjected to northern blot analysis, as described.

### qRT-PCR analysis

To eliminate any genomic DNA contaminations, the RNA samples underwent a purification step using 0.25 U of DNase I, RNase-free (Thermo Fisher Scientific, #EN0521) for every 1 µg of RNA, incubating for 45 min at 37°C. The qRT-PCR analysis was conducted utilizing the Takyon No ROX SYBR 2x Mastermix blue (Eurogentec #UF-NSMT-B0701) in accordance with the guidelines provided by the manufacturer, employing a QuantStudio 5 Real-Time PCR System (Thermo Fisher Scientific) for detection.

## Results

### Establishment of RIL-seq for *Salmonella* inside macrophages

In order to globally map RNA–RNA interactions in an intracellular pathogen, we established Hfq RIL-seq for intramacrophage *Salmonella* (Fig. 1A). Previous RIL-seq experiments were typically performed on >4 × 10^10^ bacterial cells [35, 40], a quantity far too large to be reasonably recovered from host cells under standard infection conditions. Therefore, we systematically determined the minimal number of bacteria necessary to immunoprecipitate RNA with FLAG-tagged Hfq from *Salmonella* cells (Supplementary Fig. S1A). Our analysis showed that 10^7^ bacteria are sufficient for ready detection of Hfq by western blot, while a faint Hfq band was visible even for 10^6^ bacteria. Although an immunoblot signal alone may not strictly define the lower limit for successful RIL-seq, we used this as a practical benchmark for immunoprecipitation performance under our conditions. To determine the infection conditions necessary to reach these cell numbers, we incubated murine RAW264.7 macrophages (an established host model [47, 48]) with opsonized *Salmonella*, which promotes bacterial engulfment. CFU counts of the recovered intracellular bacteria at defined time points suggested that the minimally required bacterial cell number is reached at 20 h p.i. at a MOI of 50 (Supplementary Fig. S1B).

When we subjected the infection samples to RIL-seq, we found that direct lysis, without prior depletion of host-derived material (“lysis strategy 1” in Supplementary Fig. S1C), resulted in few bacterial reads (∼10% of total reads; Supplementary Table S1). We therefore used a different harvesting protocol [49] in which the host cells are selectively lysed with a mild detergent, followed by centrifugation of the released bacteria (“lysis strategy 2” in Supplementary Fig. S1C). Although this extra step reduced the amount of recovered Hfq protein (Supplementary Fig. S1D), it efficiently decreased the host read fraction and concomitantly increased the proportion of reads that mapped to the *Salmonella* genome to ∼34% (Supplementary Table S1). What is more, this bacterial enrichment prior to RIL-seq also yielded more chimeric reads (from 1.7% with strategy 1 to almost 10% of the *Salmonella*-mapped reads with strategy 2; Supplementary Table S1). Based on these findings, we chose selective lysis (strategy 2) for our subsequent intramacrophage RIL-seq experiments.

We performed intramacrophage RIL-seq on FLAG-tagged Hfq in two biological replicates and sequenced the co-purified RNA to a depth of 60–130 million paired-end reads per library (Supplementary Table S1). Samples from macrophages infected with wild-type bacteria expressing only untagged Hfq served as controls. The distribution and relative abundance of each RNA type associated with Hfq fitted well with previous Hfq RIL-seq studies of bacteria grown *in vitro* [30, 35]. Specifically, sRNAs, mRNAs, and untranslated regions (UTRs) dominated the chimeras in the *hfq::3xFLAG* strain (Fig. 1B). As expected, most of the chimeric fragments were between sRNAs and 5′UTRs and between sRNAs and CDSs (Fig. 1C). Hfq-bound sRNAs are oftentimes strongly engaged with the proximal face of the Hfq hexamer through their 3′ poly(U) tails, rendering them less likely to undergo proximity ligation, particularly when the sRNA is not simultaneously interacting with a target RNA [40]. Accordingly, our *in vivo* dataset recapitulated this positional bias, with 5′UTRs and CDSs enriched at the first, and sRNAs at the second position of chimeras [35, 40] (Fig. 1D). Reassuringly, the most abundant sRNAs on Hfq in intracellular *Salmonella* were PinT and MgrR (rank 1 and 2, with ∼18.5% or ∼9.8% of all sRNA-mapped reads, respectively), both of which are activated by the PhoP/Q system [18, 50] (Fig. 1E).

### Intramacrophage RIL-seq correlates with conventional RIL-seq under SPI-2-inducing conditions, yet also highlights *in vivo*-specific interactions

The intramacrophage environment can be partially recapitulated *in vitro*, through batch growth of *Salmonella* in synthetic medium designed to mimic the vacuolar milieu, referred to as “SPI-2 medium” [51]. For comparison with the above *in*-*vivo* data, we applied the conventional RIL-seq protocol [35] to *Salmonella* growing in SPI-2 medium (Supplementary Fig. S2). Our intramacrophage RIL-seq approach is validated by the fact that when performed under two similar conditions (intracellularly and in SPI-2-inducing minimal medium), the most abundantly purified sRNAs and their interactomes look very similar. For example, MgrR, PinT, and ChiX were the three top-enriched sRNAs in both datasets, although their relative contribution to the chimeric fragments varied (compare Fig. 1E and Supplementary Fig. S2D).

Having said this, we would not expect the SPI-2 medium to fully recapitulate the complexity of the intracellular environment, the latter of which includes dynamic host-derived signals, nutrient limitation, and immune pressures [52]. Indeed, we can see several differences between the two datasets. For example, MgrR sRNA is an established repressor of targets involved in LPS modification [50, 53]. In both our RIL-seq datasets, *pitA*, encoding an inorganic phosphate transporter, emerged as an additional MgrR target (Supplementary Fig. S3A  and B). Conversely, only the intramacrophage RIL-seq data detected the *rpoS* mRNA encoding the stationary phase sigma factor, σ^S^, as another potential MgrR target (Supplementary Fig. S3A). Intriguingly, the MgrR-binding site overlaps the *rpoS* 5′ region targeted by the activating sRNAs ArcZ, DsrA, and RprA [54, 55]. Notably, while MgrR and *pitA* expression levels are comparable between both conditions, *rpoS* expression is higher inside macrophages compared with the SPI-2 *in vitro* condition [52]. As another example, the abundant sRNA ChiX represses *chiP*, encoding an outer membrane protein involved in the uptake of chitin-derived oligosaccharides [56]. Our RIL-seq data predicted several additional targets for this sRNA: e.g. the *ais* mRNA, encoding a hypothetical protein induced by aluminum, was recovered in ChiX chimeras under both conditions, i.e. inside macrophages and *in vitro* (Supplementary Fig. S3A and B). In contrast, high-ranking RIL-seq interactions that were unique to the macrophage dataset included chimeras between ChiX and the *yfhp* and *ftsE* mRNAs (red in Supplementary Fig. S3A). Of those, *yfhp* encodes the predicted DNA–binding transcriptional regulator IscR and its expression is induced inside macrophages, yet not in the *in*-*vitro* SPI-2 condition [52]. FtsE is a cell division protein, whose mRNA is constitutively—albeit lowly—expressed [52]. While validation of these newly predicted target candidates is pending, these observations indicate that the intramacrophage RIL-seq might uncover regulatory interactions that would otherwise escape detection, either due to a low abundance of the target mRNA (*rpoS, yfhp*) or due to conditional requirements not reconstituted in the *in vitro* situation (*ftsE*).

To facilitate the exploration of our data by the public, we have launched an intuitive open-access online browser that is available under https://resources.helmholtz-hiri.de/rilseqset2025/. Here, users can interact with our RIL-seq datasets in a condition-specific manner, visualize the complex RNA–RNA networks, and search for specific genes and sRNAs to display their target interactions. For each pair, the corresponding interaction sites can also be visualized within their genomic context.

### InvS is a PhoP-induced 3′-derived sRNA that accumulates in intracellular *Salmonella*

Given the dominance of PinT reads in both the intramacrophage and SPI-2 RIL-seq data, we reasoned that these datasets could point to potential negative regulators of this sRNA (as motivated in the Introduction). While more chimeric fragments involving PinT were detected under SPI-2-inducing conditions compared to the intramacrophage environment, a substantial overlap was observed: 17 out of 25 interactors detected under SPI-2 conditions were also found inside macrophages (Supplementary Fig. S3C and D), including several established PinT targets such as *grxA* [18] and *steC* [21]. Importantly, both datasets differed markedly from the PinT interactome from a previous *Salmonella* Hfq RIL-seq study under a SPI-1-related condition [35] (Supplementary Fig. S3E). Searching for PinT chimeras with other sRNAs, we captured 19 or 13 putative PinT–sRNA pairs in these two datasets, respectively. The most frequent sRNA interactor of PinT in both conditions was InvS (pie charts in Fig. 1E and Supplementary Fig. S2D). Interestingly, InvS was also amongst the top-five transcripts in a previous MS2-affinity purification coupled with RNA sequencing (MAPS) study, in which we sequenced RNA interactors following co-purification with aptamer-tagged PinT sRNA from *Salmonella* grown in SPI-2 medium [21]. Therefore, we chose to pursue InvS’s role in *Salmonella* virulence and as a likely candidate for an RNA antagonist of PinT.

**Figure 2.**
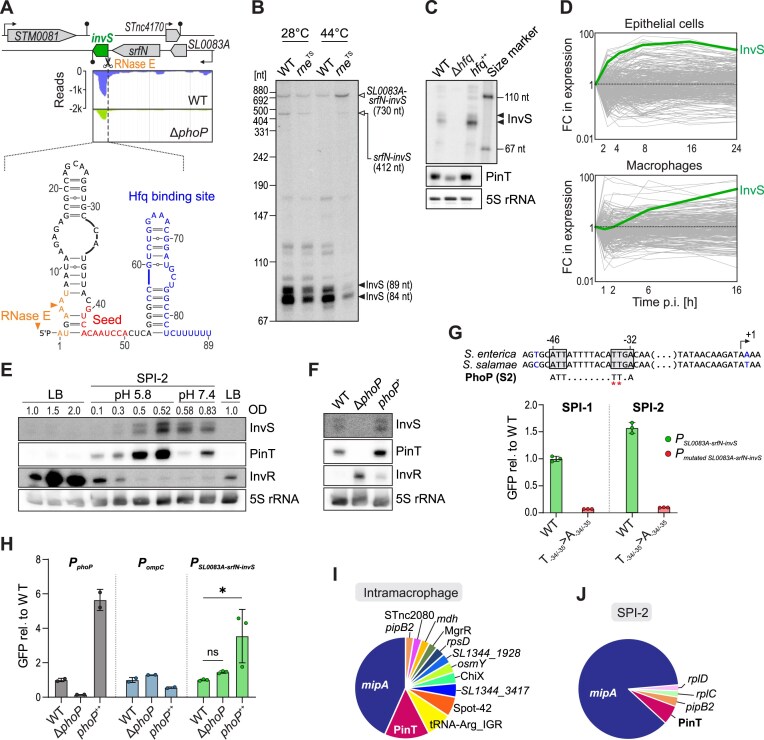
InvS expression, biogenesis, and interactome. (**A**) InvS genomic location and structure. RNA secondary structure predicted *in silico* by mFold software. The Hfq-binding site highlighted is based on previously published CLIP-seq data [15]. Information on RNase E cleavage sites stems from [59]. The seed region is determined in the present study (see Fig. 3). RNA-seq data mapped to the *SL0083A-srfN-invS* operon in a PhoP-deficient *Salmonella* mutant and its isogenic wild-type strain is derived from [61]. (**B**) InvS expression levels in a temperature-sensitive RNase E mutant (*rne*^TS^) and wild-type *Salmonella*. Northern blot showing RNA extracted from the respective overnight cultures grown at 28°C. Cultures were subsequently shifted to 44°C for 30 min before RNA purification. The positions of the two mature InvS isoforms (84 and 89 nt) are indicated by black arrowheads and that of the precursor transcripts, namely full-length *SL0083A*-*srfN*-*invS* (730 nt [52]) and *srfN*-*invS* (∼400 nt [62, 58]), by white arrowheads. (**C**) InvS expression is Hfq-dependent. RNA was extracted from the indicated *Salmonella* strains grown in SPI-2 medium overnight and analyzed by northern blotting. PinT, which is known to be Hfq-dependent [13], was loaded for comparison and 5S rRNA as the loading control. (**D**) RNA-seq data from [18] show InvS expression during infection of epithelial cells and macrophages relative to the corresponding expression levels in the bacterial inoculum. (**E**) InvS expression follows that of the *bona fide* PhoP-induced sRNA PinT and is anti-correlated with the SPI-1-associated InvR sRNA. Northern blot data from an *in vitro* SPI-1/SPI-2 transition assay. For each transition between LB and SPI-2-inducing minimal medium, the bacteria were washed and re-inoculated at a ratio of 1:50. The result is representative of three biological replicates. (**F**) InvS expression is PhoP-dependent. RNA was extracted from the indicated *Salmonella* strains grown in SPI-2 medium overnight to OD ≈ 0.4 and analyzed by northern blotting. PinT and InvR, whose transcription is known to be induced by PhoP [18] or HilD [63], respectively, were loaded for comparison and 5S rRNA was the loading control. (**G**) PhoP box is required for transcriptional activation. A –138 to +162 fragment relative to the TSS of *SL0083A-srfN-invS* operon was cloned upstream of GFP on pAS093 plasmid (termed *P_SL0083A-srfN-invS_*), along with a mutant version containing point mutations (T-34/-35 → A-34/-35) in the putative PhoP box. Constructs were transformed into wild-type (WT) *Salmonella* and GFP expression in LB medium was measured by flow cytometry, showing the PhoP box is essential for transcriptional activation of this operon. (**H**) Validation of PhoP-dependent regulation using GFP reporter constructs. The GFP reporter construct from Fig. 2G was transformed into WT, Δ*phoP*, and *phoP*⁺⁺ strains (green). For comparison, GFP reporter constructs carrying the *phoP* promoter (positive control, black) or *ompD* promoter (negative control, blue) were also introduced into the same strains. The cultures grew in LB reaching OD = 2.0 and the GFP levels were measured by flow cytometry. For the positive and negative controls, two replicates were included; for *P_SL0083A-srfN-invS_*, three biological replicates were included and significance was tested using ordinary one-way ANOVA with Dunnett’s multiple comparisons test. ns: *P_adj_* > 0.05, *: *P_adj_* ≤ 0.05. (**I** and **J**) InvS interactome inside macrophages (I) and under SPI-2-inducing conditions (J), respectively, based on RIL-seq.

InvS (aka STnc470) is a ∼90 nt-long, Hfq-associated sRNA [15, 57]. The InvS sequence is well conserved across *Salmonella* species (Supplementary Fig. S4A) but not present in other members of the *Enterobacteriaceae*. InvS was shown to promote *Salmonella* invasion of HeLa cells [58], yet its biogenesis and direct targets have remained elusive. InvS has been annotated as a 3′ end-derived sRNA since it overlaps with the 3′UTR of the coding gene *srfN* (aka *STM0082*; encoding a probable secreted protein). Given the absence of a transcriptional start site (TSS) in the intergenic region between *srfN* and *invS*, but the presence of two predicted RNase E cleavage sites [59]—one coinciding with the adenine at the annotated 5′ end of the sRNA and an alternative cleavage site (AA↓AU, +4 to + 7)—we hypothesized that InvS is generated through ribonuclease processing of the polycistronic *SL0083A*–*srfN*–*invS* transcript (Fig. 2A). In support of this hypothesis, we observed two distinct InvS bands on a northern blot, which both stopped accumulating when the major endoribonuclease RNase E was inactivated in an established temperature-sensitive *Salmonella* RNase E mutant strain (*rne*^TS^; [56, 60]) (Fig. 2B). Accordingly, the previously annotated InvS isoform of 89 nt likely corresponds to the upper band, whereas a slightly shorter isoform of ∼84 nt arises from cleavage at this alternative position. Northern blot analysis further demonstrated that InvS expression is strongly reduced in the absence of Hfq and restored upon complementation (Fig. 2C), confirming that InvS accumulation is Hfq-dependent.

Interrogation of existing transcriptomics data [61] suggested reduced expression of the *SL0083A*–*srfN*–*invS* operon in a *phoP*-deficient *Salmonella* mutant strain relative to wild-type bacteria (Fig. 2A). In addition, while barely detectable in extracellular *Salmonella*, InvS strongly accumulated after invasion of epithelial cells or upon uptake of *Salmonella* by macrophages [18, 52] (Fig. 2D). In other words, InvS expression echoed that of the *bona fide* PhoP-induced sRNA PinT (Supplementary Fig. S4B). Using an *in vitro* SPI-1 to SPI-2 transition assay, we could reconstitute these expression profiles, confirming that InvS expression follows the kinetics of PinT induction (Fig. 2E). Northern blot probing confirmed that a Δ*phoP* mutation reduced InvS expression, while *trans*-complementation of the PhoP transcription factor (*phoP*^++^) fully restored InvS sRNA levels to that of the wild-type, both very similar to PinT (Fig. 2F). We also noticed a potential PhoP box (subclass S2 according to the classification of [64]) ∼35 bp upstream of the *SL0083A*–*srfN*–*invS* operon’s annotated TSS (Fig. 2G, upper). We thus generated a transcriptional reporter, cloning the region –138 to + 162 relative to the TSS upstream of a green fluorescent protein (GFP) reporter cassette on a plasmid. A transcriptional fusion with two point mutations in the putative PhoP box (T_-34/-35_ to A_-34/-35_) was made to serve as control. These reporters were inserted in wild-type *Salmonella* as well as in *phoP* deletion and overexpression strains. Using GFP fluorescence as a quantitative readout, these reporters revealed that the thymines at positions –34 and –35 are essential for transcriptional activation (Fig. 2G). Additionally, the data confirmed that PhoP enhances transcription of the *SL0083A*–*srfN*–*invS* operon; however, activation also occurred in the absence of PhoP, indicating additional regulatory factors are at play (Fig. 2H). Together, these data suggest that InvS biogenesis is dependent on both, PhoP-mediated transcriptional activation and RNase E-catalyzed terminal cleavage of the *SL0083A*–*srfN*–*invS* primary transcript.

### InvS represses the host-adhesion protein MipA by occluding its translation initiation region

**Figure 3.**
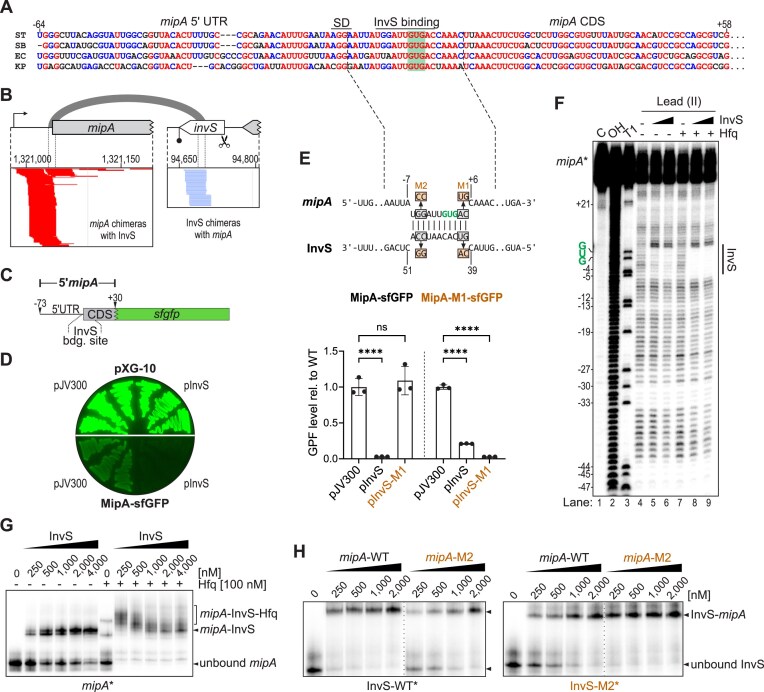
InvS represses MipA by occluding the translation initiation region of the mRNA. (**A**) Sequence alignment indicates that the InvS-binding site on *mipA* mRNA is conserved among *S. enterica* serovar Typhimurium (ST), *S. bongori* (SB), *E. coli* (EC), and *K. pneumonia* (KP). SD refers to Shine-Dalgarno sequence. Numbers are relative to the translation start codon (green; +1 position). (**B**) Genome browser-derived screenshot of the stacked reads of chimeric fragments mapping to the InvS sRNA and *mipA* mRNA (from replicate 1 of the intramacrophage RIL-seq). Reads corresponding to the first or second position in the chimera are colored red or blue, respectively. The gray loop illustrates the inter-molecular base-pairing region (see panel E, upper) relative to the chimeric fragments. (**C**) A low-copy plasmid was constructed to carry the *sfgfp* gene under the control of a region spanning the 5′UTR and the first 10 amino acids of *mipA* open reading frame (–73 to +30, relative to the start codon). This region was selected based on a predicted putative binding site for the InvS sRNA on the *mipA* mRNA. (**D**) LB-agar plate showing the fluorescence of streaked strains containing the fluorescence control plasmid (pXG-10) with either overexpression of InvS (pInvS) or the empty vector (pJV300) in the upper section and the corresponding strains containing the translational MipA–sfGFP fusion below. (**E**) Upper: IntaRNA-predicted base-pair interaction between *Salmonella* InvS and the translation initiation region of *mipA*, with nucleotides mutated for the below fluorescent reporter assay (“M1”) and the EMSA in panel H (“M2”) depicted in orange boxes. Lower: fluorescence quantification of MipA–sfGFP translational fusion by flow cytometry. Strains carried either the wild-type fusion or the mutant variant (MipA-M1; AC→UG) together with either the empty vector (pJV300), the InvS-WT, or InvS-M1 overexpression plasmid. Data are presented as mean ± SD from three independent biological replicates. Statistical significance was assessed using two-way ANOVA with Dunnett’s multiple comparisons test relative to the corresponding control. ns: *P_adj_* > 0.05, ****: *P_adj_* < 0.0001. (**F**) Structure probing experiment was performed using a 5′ end-radiolabeled *mipA* RNA fragment containing the predicted InvS-binding site. The fragment was incubated with increasing concentrations of unlabeled InvS sRNA, in the presence or absence of purified Hfq protein. (**G**) EMSA with radioactively labeled *mipA* RNA fragment was performed to assess the binding interaction between InvS sRNA and the *mipA* transcript in absence and presence of Hfq. (**H**) EMSA of radiolabeled InvS (wild-type [WT] or mutated [M1]) with increasing concentrations of the *mipA* mRNA fragment or a compensatorily mutated version thereof. The respective mutations are indicated in panel (E), upper.

RIL-seq detected InvS as a major interactor of PinT; we next investigated whether PinT likewise interacts strongly with InvS. Intriguingly, when inspecting the InvS-harboring chimeras, PinT only scored second, with *mipA* mRNA being highlighted as the most frequent RNA interactor in both the intramacrophage and SPI-2 datasets (Fig. 2I and J). The inferred interaction involved the region between nucleotides 40 and 50 of InvS (relative to its 5′ end; Fig. 2A) and the translational start codon of *mipA* (Fig. 3A and B). This would suggest that InvS acts as a translational repressor of the outer membrane protein, MipA. To test this hypothesis, we constructed a low-copy plasmid carrying the 5′ portion of *mipA* from the transcriptional start site to the 10th codon (nucleotide positions –73 to + 30 relative to the GUG start codon) fused to the open reading frame of superfolder-GFP (sfGFP, [46]) (Fig. 3C). Indeed, when introduced into *Salmonella*, this MipA-sfGFP translational fusion produced a high fluorescent signal, which was completely lost upon co-expression of the InvS sRNA from a second plasmid (Fig. 3D). To validate the predicted base-pairing interaction, point mutations were introduced into the InvS-binding site of *mipA*, immediately downstream of the start codon (AC→UG; *mipA*-M1), together with compensatory mutations in InvS (InvS-M1) (Fig. 3E). As expected, InvS-M1 was unable to repress the wild-type MipA-sfGFP. Inversely, wild-type InvS retained the capability to suppress *mipA*-M1, albeit less effectively than *mipA*-WT. Importantly, full repression was restored when InvS-M1 was co-expressed with *mipA*-M1, supporting the predicted base-pair interaction.

Next, we performed *in vitro* biochemical assays to resolve the direct binding between InvS and the *mipA* translation initiation region at the nucleotide level. First, a structure probing experiment (Pb^2+^-induced cleavage) was conducted, incubating a 5′ end-radiolabeled *mipA* fragment containing the predicted InvS-binding site with increasing concentrations of unlabeled InvS in the presence or absence of purified Hfq protein. In line with the chimeric fragments obtained from RIL-seq, InvS clearly protected from cleavage at the region around the GUG start codon of *mipA* (Fig. 3F). While this protection was observed even without Hfq, the presence of this RNA chaperone in the reaction mix enhanced the intensity of the footprint. Second, Hfq also increased the binding affinity of InvS to the *mipA* mRNA fragment in an electrophoretic mobility shift assay (EMSA), with the dissociation constant (*k*_d_) decreasing from 1 µM without Hfq to <250 nM with Hfq (Fig. 3G). Importantly, the introduction of two point mutations (“InvS_M2”; Fig. 3E, upper) in the putative seed region of InvS reduced target binding, but binding was fully restored when introducing two compensatory mutations in the *mipA* fragment (Fig. 3H). Collectively, these experiments indicate that InvS sequesters the start codon of the *mipA* mRNA and thereby inhibits MipA synthesis. While occurring even in the absence of an assisting protein under optimal conditions in a test tube, the InvS-*mipA* interaction is enhanced by Hfq.

### Seed region interaction of InvS and PinT reduces InvS stability

**Figure 4.**
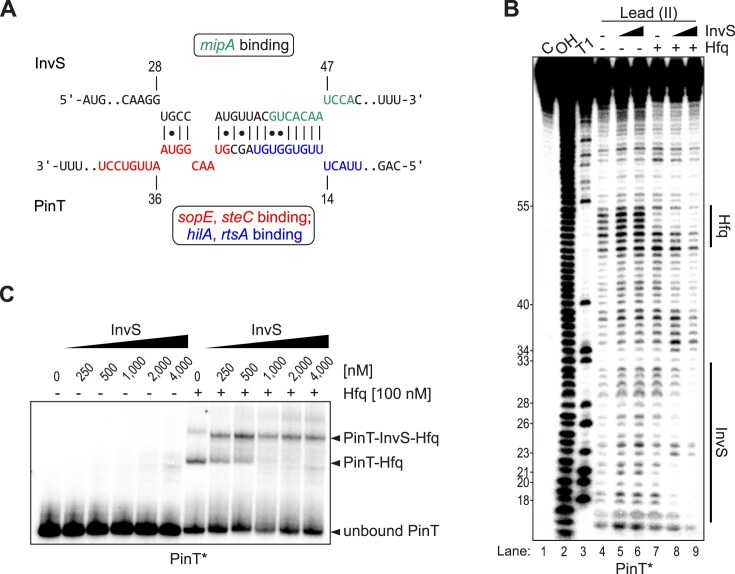
The two sRNAs InvS and PinT form an intermolecular duplex in the presence of Hfq. (**A**) IntaRNA prediction of duplex formation between InvS and PinT. The respective seed regions of both sRNAs are colored. Note that PinT uses distinct seed regions to bind SPI-1 transcription factor mRNAs, *hilA* and *rtsA* [20] (blue), and to interact with effector-encoding mRNAs [18, 21] (red), all of which are partially occluded within the InvS–PinT duplex. (**B**) The protected PinT region corresponds to the predicted InvS-binding site (see panel A). The second protected region near the 3′ end of PinT corresponds to the Hfq-binding site [15]. (**C**) Hfq facilitates the formation of a stable PinT–InvS complex. EMSA with 5′-end labeled PinT showed a stable complex with cold InvS, only after pre-incubation with Hfq, as indicated by a gel shift.

The second-ranked ligand of the InvS sRNA in the RIL-seq data was PinT (Fig. 2I and J). *In silico* prediction using the IntaRNA tool [65, 66] suggested that InvS and PinT may form a duplex in which the putative seed region of either sRNA would be partially occluded (Fig. 4A). We therefore probed the predicted InvS-PinT interaction *in vitro*, incubating 5′ end-labeled PinT with increasing concentrations of unlabeled InvS (Fig. 4B). InvS protected PinT from Pb^2+^-induced cleavage, but only in the presence of Hfq. Similarly, in an EMSA, 5′ end-labeled PinT was only shifted by InvS when the two RNAs were pre-incubated with Hfq (Fig. 4C). Collectively, these results imply that—at least under *in vitro* conditions—the two Hfq-dependent sRNAs, InvS and PinT, form a complex on Hfq through recognizing each other’s seed sequence.

**Figure 5.**
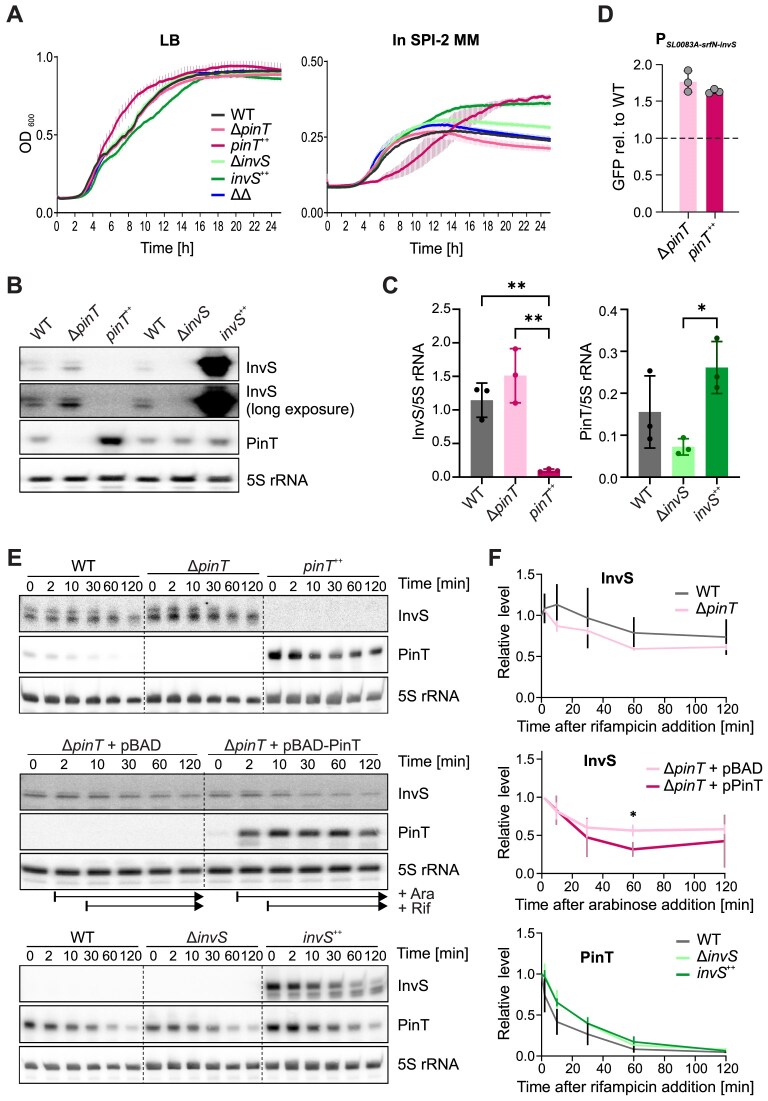
PinT reduces InvS stability. (**A**) Growth curves of the indicated *Salmonella* sRNA deletion and overexpression strains relative to their parental wild-type (WT) under the SPI-1- (LB, OD_600_ 2.0) or SPI-2-inducing condition (SPI-2 minimal medium, OD_600_ 0.3). (**B** and **C**) Northern blot-based measurement of InvS and PinT steady-state levels in the indicated strain backgrounds grown in SPI-2-inducing medium. Panel B shows a representative result out of three biological replicates per strain that were grown in SPI-2 medium overnight, and panel C shows the quantification over all replicates. Statistical significance was assessed using one-way ANOVA with Tukey’s multiple comparisons test. *: *P_adj_* ≤ 0.05, **: *P_adj_* ≤ 0.01. (**D**) Transcriptional reporter assay demonstrating that the transcriptional activity of the *SL0083A*-*srfN*-*invS* operon is independent of PinT levels. Data represent the mean ± SD from three biological replicates (individual gray dots). (**E** and **F**) Rifampicin-mediated RNA-stability assay. At *t*_0_, *de novo* transcription was halted and transcript decay traced over time by northern blotting. In each case, a representative northern blot out of 4 (upper blot), 3 (middle), or 6 (lower) independent replicate experiments is shown in panel E, and the corresponding quantifications in panel F. The asterisk indicates a statistically significant difference in relative InvS levels between the strain harboring the PinT expression plasmid and that containing the empty control vector at 60 min after halting *de novo* transcription (*: *P* = 0.0292; unpaired *t*-test with Welch’s correction).

We next addressed the consequences of the PinT–InvS interaction *in vivo*, i.e. asking whether one sRNA regulated the other. For functional assays, we harnessed *Salmonella* strains lacking the *invS* or the *pinT* gene alone, or in combination (double knockout dubbed “ΔΔ”). In line with our previous findings [18], *pinT* deletion did not influence *Salmonella* replication *in vitro* (Fig. 5A). Similarly, deletion of *invS* alone or in conjunction with *pinT* did not impact on *Salmonella* growth. We also constructed strains overexpressing either PinT or InvS from a constitutive promoter on a high-copy plasmid. In LB medium, the PinT overexpression strain (*pinT*^++^) exhibited faster growth compared to the other strains, whereas InvS overexpression (strain *invS*^++^) slowed growth. In the SPI-2 medium, *pinT*^++^ had a longer lag-phase than the other strains, while both sRNA overexpression strains accumulated to higher cell densities than wild-type bacteria (Fig. 5A). Importantly, northern blot probing of total RNA extracted from the respective strains grown in SPI-2 medium showed elevated steady-state levels of InvS in the Δ*pinT* background, whereas PinT overexpression fully depleted InvS (Fig. 5B and C).

To explore the cause of PinT’s impact on InvS levels, we conducted several experiments. First, using transcriptional reporter strains, we ruled out a negative influence of PinT overexpression on the promoter that drives transcription of the *SL0083A*–*srfN*–*invS* locus (Fig. 5D). Second, rifampicin-mediated transcription runout experiments in SPI-2-inducing medium found no effect of deleting *pinT* on InvS stability (Fig. 5E and F, upper panels). As shown above, constitutive overexpression of PinT fully depleted InvS, this preventing half-life determination of the latter. We therefore opted for an inducible expression system, which allowed us to turn on PinT synthesis for 2 min prior to the addition of rifampicin. This revealed a PinT-dependent reduction of the half-life of InvS, as compared to an analogously treated strain harboring the empty control vector (Fig. 5E and F, middle panels). We conclude that PinT lowers InvS expression, at least partially through accelerated decay. In contrast, *invS* deletion or overexpression had little impact on PinT steady-state levels (Fig. 5B and lower panels in Fig. 5E and F). Obviously, this does not exclude the possibility that InvS may affect PinT activity; a hypothesis which we address below.

### PinT-suppressed virulence factors are de-repressed by InvS, while PinT counteracts MipA repression

**Figure 6.**
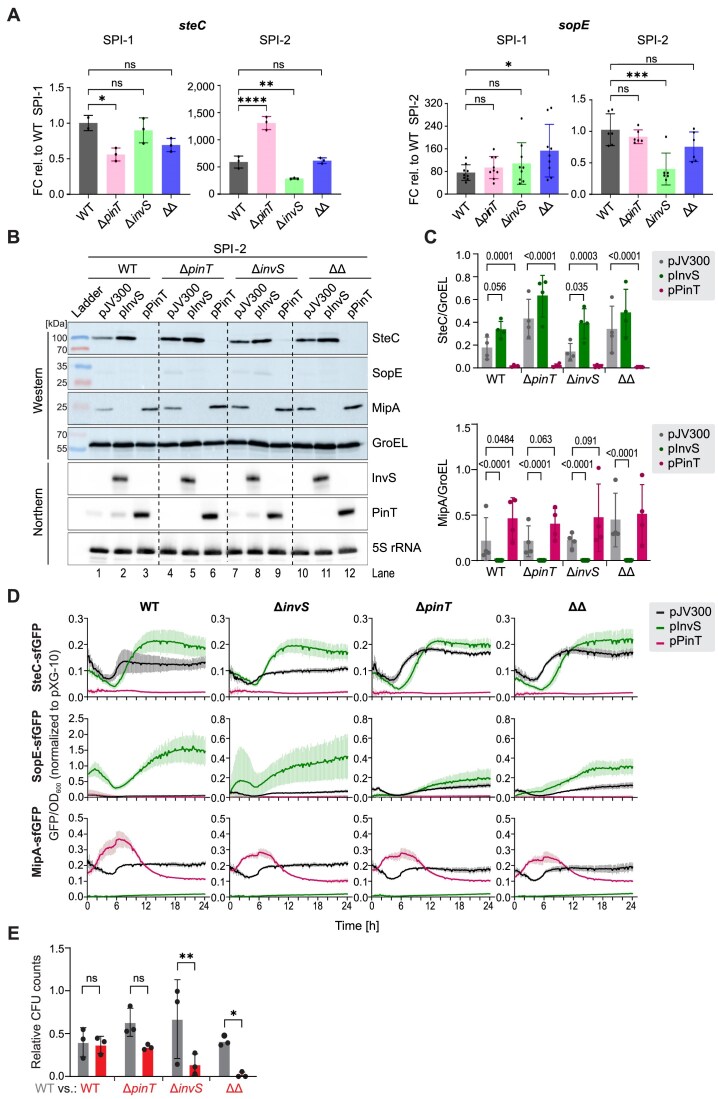
PinT and InvS mutually affect each other’s target genes and impact on *Salmonella* fitness inside macrophages. (**A**) Expression analysis of PinT targets. Quantitative real-time PCR (qRT-PCR) was performed to measure the expression levels of *sopE* and *steC* (encoding a SPI-1 and SPI-2 effector protein, respectively) in wild-type (WT), Δ*pinT*, Δ*invS*, and ΔΔ strains. The RNA samples were collected under SPI-1- (LB, OD = 2.0) and SPI-2-inducing conditions (SPI-2 MM, OD = 0.3). The quantification cycle (Cq) values were normalized to those of the reference gene 5S rRNA. Data represent the mean ± SD from ≥3 biological replicates (shown on bars by black dots), each with ≥3 technical replicates. Statistical significance was determined using one-way ANOVA with Dunnet’s multiple comparisons test. ns: *P_adj_* > 0.05, *: *P_adj_* ≤ 0.05, **: *P_adj_* ≤ 0.01, ***: *P_adj_* ≤ 0.001, ****: *P_adj_* < 0.0001. (**B** and **C**) Western blot analysis of endogenous MipA, SopE-FLAG, and SteC-FLAG protein levels under the SPI-2 condition. Western blot was performed using a custom antibody raised against endogenous MipA (24 kDa) and a strain with chromosomally FLAG-tagged C-termini of endogenous SopE (26.6 kDa) and SteC (44.3 kDa). Protein samples were collected from the SPI-2-inducing condition and separated by SDS–PAGE, followed by immunoblotting with anti-MipA and anti-FLAG antibodies. GroEL was used as a loading control and the integrity of the strains was confirmed by corresponding northern blots of total RNA extracted from the same cultures. Panel B shows a representative result from three independent biological replicates and panel C the quantification over all replicates. Note that the low SopE levels under this condition prevented its robust quantification. Statistical significance was determined using lognormal ordinary one-way ANOVA and *P*-values < 0.1 are plotted. For corresponding western blot data under the SPI-1 condition, see Supplementary Fig. S5. (**D**) Dual plasmid reporter assay of InvS and PinT targets in defined mutant backgrounds. Each background harbors an InvS or a PinT expression plasmid, or the empty vector control (pJV300). Data derive from three biological, with each two technical replicates. OD_600_ and fluorescence intensities were read out in a plate reader for 24 h. GFP fluorescence was first normalized by calculating the GFP/OD_600_ ratio for each well (Supplementary Fig. S6), followed by a second normalization to the mean GFP/OD ratio of the pXG10 control (set to 1). Note the differing Y-axis range in the first SopE-sfGFP graph, since the fluorescence intensity of the WT strain co-expressing InvS and SopE-sfGFP was markedly higher than that of the other strains. (**E**) Competitive fitness of *Salmonella* sRNA mutant strains during intracellular infection. WT and the indicated mutant strain were mixed in a 1:1 ratio and the resulting mixture was opsonized and used as the inoculum to infect RAW264.7 macrophages at a total MOI of 25. At 20 h p.i., the competitive fitness of the strains was deduced from strain-specific CFU counting, normalized to the respective inoculum. Data are derived from three independent biological replicates, each comprising two technical replicates, and statistical significance was determined using two-way ANOVA. ns: *P *> 0.05, *: *P *≤ 0.05, **: *P *≤ 0.01.

We next evaluated whether InvS and PinT could modulate each other’s regulatory activities (e.g. via sponging). To this end, we introduced either a control plasmid, an InvS-overexpression plasmid, or a PinT-overexpression plasmid into four genetic backgrounds (wild-type, Δ*pinT*, Δ*invS*, and Δ*pinT*Δ*invS*) and monitored established sRNA targets on RNA and protein level. The rationale was that indirect effects of overexpressing an sRNA that depend on the presence of the respective other sRNA would be lost in the corresponding deletion backgrounds. Performing quantitative real-time PCR (qRT-PCR), we observed that target mRNAs accumulated similarly in the different mutant strains and the wild-type in a SPI-1-inducing condition (LB, OD_600_ = 2.0) (Fig. 6A, “SPI-1”). Expression changes were more pronounced in the SPI-2 medium (Fig. 6A, “SPI-2”), as expected given that both endogenous sRNAs are maximally induced in the latter medium. Particularly, the absence of InvS decreased the levels of both tested PinT target mRNAs encoding the effector proteins SopE [18] and SteC [21]. However, qRT-PCR failed to reliably quantify the levels of the InvS target, *mipA* mRNA. Therefore, we produced a polyclonal antiserum against endogenous MipA for immunoblotting. This antiserum, in combination with a previously constructed *Salmonella* strain in which the C-termini of endogenous SopE (26.6 kDa) and SteC (44.3 kDa) are both FLAG-tagged in the chromosome [18], allowed us to detect all three proteins of interest in one sample. Western blot analysis under the SPI-1 (Supplementary Fig. S5) and SPI-2 (Fig. 6B and C) conditions revealed a slight increase in MipA protein levels by overexpressing PinT (e.g. compare lanes 3 to 1 in Fig. 6B). InvS overexpression under the SPI-1 condition not only entailed the downregulation of its cognate target protein, MipA, but also reduced the levels of SopE, whereas SteC was not affected (Supplementary Fig. S5). In contrast, immunoblotting under the SPI-2-inducing condition (i.e. when endogenous PinT is highly expressed) revealed that overexpression of InvS increases SteC protein levels (SopE protein could not be reliably detected under the SPI-2 condition) (Fig. 6B and C). In the case of SteC, the positive effect of InvS was largely dependent on the presence of endogenous PinT (see lanes 2 versus 1 and 8 versus 7 in Fig. 6B and corresponding *P*-values in Fig. 6C).

To investigate the temporal dynamics of sRNA-mediated regulation in the presence or absence of the respective sRNA antagonist, we monitored fluorescence from existing translational reporter constructs based on sfGFP. *Salmonella* strains harboring either MipA–sfGFP, SopE–sfGFP, or SteC–sfGFP were grown for 24 h in SPI-2 medium and cell density (OD_600_) and fluorescence intensity were measured in 10 minutes intervals (Fig. 6D and Supplementary Fig. S6). Consistent with our earlier observations, the MipA reporter (Fig. 3C,D) was again strongly repressed by overexpression of InvS (Fig. 6D). PinT overexpression accelerated the accumulation of MipA–sfGFP, albeit this effect seemed independent of endogenous InvS in this assay. As expected [21], SteC–sfGFP fluorescence was repressed by PinT (Fig. 6D). In line with the observations made on mRNA and protein levels in the SPI-2 medium (Fig. 6A–C), InvS induction led to the de-repression of SteC, with the effect being more pronounced in the presence of endogenous PinT (Fig. 6D). Lastly, SopE–sfGFP accumulated when InvS was overexpressed or *pinT* deleted (Fig. 6D), indicating that in SPI-2-inducing conditions, InvS counteracts PinT-mediated repression of SopE. Notably, the positive influence of InvS on SopE accumulation was markedly reduced in the absence of endogenous PinT, in line with a PinT-sponging effect of InvS.

### Evaluation of the conditional regulation of SopE and as a direct InvS target candidate

In light of the negative effect that InvS overexpression exerted on SopE protein levels under the SPI-1 condition (Supplementary Fig. S5), we asked whether InvS could also directly target the *sopE* mRNA. Although no *sopE*-InvS chimeras were detected under any of the previous [35] or current (Fig. 2I and J) RIL-seq conditions, a targeted *in*-*silico* analysis suggested potential base-pairing of InvS with the translation initiation region of *sopE*, through the same region that recognizes *mipA* (Supplementary Fig. S7A). However, reporter assays leveraging the SopE–sfGFP translational fusion excluded a direct effect of InvS on SopE expression (Supplementary Fig. S7B). Likewise, EMSAs of a radioactively labeled *sopE* 5′ fragment incubated with increasing concentrations of InvS did not support an interaction, regardless of the presence of Hfq (Supplementary Fig. S7C). Thus, while the negative effect of ectopic InvS expression on SopE under the SPI-1 condition cannot be explained by PinT, *sopE* is also unlikely to be a direct InvS target.

We then asked whether the positive effect of InvS on SopE under the SPI-2 condition (Fig. 6A and D) is primarily due to sponging PinT, or if InvS could also titrate Hfq away from PinT-*sopE* RNA duplexes. Performing competitive EMSAs in the presence of Hfq, we indeed observed that increasing InvS concentrations sequester Hfq; this was accompanied by the depletion of the Hfq protein from its complex with PinT and *sopE* and an increase of the fractions of PinT-*sopE* dimers and free *sopE* mRNA (Supplementary Fig. S7D). We conclude that the positive influence of InvS on SopE under the SPI-2 condition is mostly through sequestration of PinT, although we cannot rule out the possibility that titration of Hfq away from the PinT-target mRNA duplexes may contribute to relieving target repression.

### InvS contributes to *Salmonella* fitness inside macrophages

Having uncovered a molecular function of InvS, we finally investigated its pathophysiological relevance in the context of a *Salmonella* infection. An *invS* deletion mutant was previously reported to be defective to invade epithelial cells [58], but InvS’ contribution to bacterial fitness inside macrophages has not yet been studied. To evaluate the competitive fitness of *Salmonella* sRNA mutant strains during intracellular infection, we performed competitive infection experiments of RAW264.7 macrophages. Specifically, we infected macrophages at a total MOI of 25 using 1:1 strain mixtures between wild-type bacteria and individual sRNA mutants, and quantified intracellular bacterial loads via strain-specific CFU counting at 20 hours p.i. (Fig. 6E). The control setup (WT versus WT) confirmed equal CFU counts, ruling out major confounding effects of the fluorescent proteins used to discriminate different strains. *Salmonella* mutants devoid of InvS exhibited a significant competitive disadvantage compared to wild-type, indicating that this sRNA contributes to intramacrophage fitness. The ∆*pinT* mutant also trended toward a reduced fitness, in accordance with our previous observations inside porcine macrophages [18], although this effect was not statistically significant under our present experimental condition. Finally, the double mutant (Δ*pinT*Δ*invS*) showed the strongest phenotype, suggesting additive effects for the deletion of both sRNAs. These results support the relevance of InvS, particularly in the intramacrophage context where the InvS–PinT interaction was originally identified.

**Figure 7.**
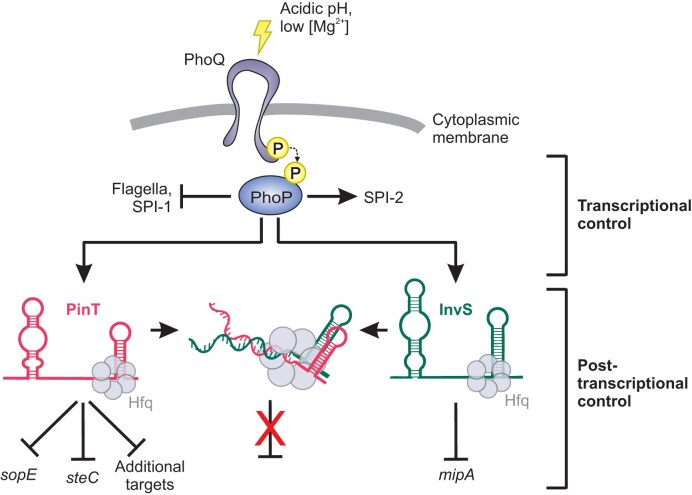
An interconnected regulatory network of two PhoP-induced sRNAs during *Salmonella* infection. Induced by intracellular cues (mildly acidic pH, low magnesium concentration), the PhoP/Q two-component system transcriptionally activates its target regulon, including the sRNAs PinT [18] and InvS (this study). In addition to the repression of their cognate direct targets (in the case of PinT, *sopE* [18] and *steC* [21] as an exemplary SPI-1 or SPI-2 gene, respectively, and *mipA* in the case of InvS), the two sRNAs associate with each other, resulting in the de-repression of their respective mRNA targets. Pointed arrows indicate activation and blunted arrows repression. For a proposed temporal model of this post-transcriptional network along *Salmonella*’s infection cycle, see Supplementary Fig. S8.

Altogether, this study suggests that inside the host environment, the PhoP-induced sRNAs PinT and InvS accumulate. Their interaction on Hfq leads to a reduction in the levels of the lesser abundant InvS sRNA and accelerates the accumulation of the InvS target MipA. *Vice versa*, sequestration of PinT by InvS elevates the protein levels of the direct PinT targets SopE and SteC (Fig. 7).

## Discussion

Along our quest to characterize the function of *Salmonella* virulence-related sRNAs, we have been focusing on PinT [67]. This sRNA is transcriptionally activated upon host cell entry through the PhoP/Q two-component system [18] and represses translation of several regulatory and effector proteins of the SPI-1 and SPI-2 regulons [18, 20, 21]. The SPI-2 virulence program is critical for *Salmonella*’s survival and replication inside the host cell vacuole. However, how PinT-mediated SPI-2 repression is relieved at later stages of infection, when this virulence program is fully activated despite PinT levels remaining high, has remained unknown. We previously speculated that intracellularly, PinT might eventually be sponged by (an)other Hfq-associated sRNA(s), thereby mitigating post-transcriptional SPI-2 inhibition [21]. However, the identification of such a postulated PinT antagonist has been hindered by a shortage of suitable methods.

RIL-seq is a powerful tool that provides a comprehensive view on a bacterium’s post-transcriptional regulatory network. Since its establishment in *Escherichia coli* [40], the method has been continuously improved and adapted to various bacterial species and experimental conditions [31, 32, 40, 68–72]. However, the relatively high input requirements have so far limited the application of the technique to large-scale *in vitro* experiments, which fail to fully replicate the complexity of natural bacterial host environments. Here, by systematically scaling down bacterial cell numbers, we reduced the input material from 4 × 10^10^ bacteria in conventional RIL-seq to ∼4 × 10^6^ (Supplementary Fig. S1B). Thanks to this 10 000-fold reduction in input material and an optimized lysis strategy, we managed to capture the Hfq-associated RNA interactome of *Salmonella* purified from infected macrophages. *Salmonella* represents a suitable model organism to establish intramacrophage RIL-seq as defined growth media exist [51, 73] that can mimic certain—albeit not all [52]—cues of the milieu of its *in*-*vivo* niche, to serve as a reference. Therefore, we were reassured, to observe a large overall similarity between our intramacrophage and SPI-2 datasets, while also identifying a few notable exceptions.

PinT emerged as a central post-transcriptional hub from both datasets. Interrogation of the RIL-seq data for PinT-interacting sRNAs highlighted InvS, an sRNA with an established virulence phenotype [58], whose molecular function was elusive. According to our RIL-seq data, the primary target of InvS was the mRNA encoding MipA (aka OmpV). Of note, MipA has an established role in mediating adhesion of *Salmonella* to epithelial cells [74]. We demonstrated here that InvS represses MipA protein synthesis by annealing to the corresponding mRNA and likely interfering with translation initiation. This seemingly creates a conundrum: how come *Salmonella* ∆*invS* mutants (that produce more MipA adhesins) are less invasive than wild-type bacteria [58]? Previously, InvS was shown to activate the expression of several SPI-1 genes, yet none of the corresponding transcripts seemed to be directly targeted by this sRNA [58]. Here, we found that the second ranked InvS interactor was the PinT sRNA, a known negative regulator of SPI-1 and SPI-2 genes [18, 20]. We showed that InvS expression relieves PinT-mediated repression of both a SPI-1 (*sopE*) and SPI-2 (*steC*) target transcript, and the same might be true for additional PinT targets such as the mRNAs for the SPI-1 activators HilA and RtsA (see Fig. 4A), thereby reconciling these observations. Our study further revealed the existence of two distinct, RNase E-generated InvS isoforms that differ by the presence or absence of five nucleotides at their 5′ end. In the future, a comparative analysis of the two isoforms could provide insights into their functional differences, such as whether truncating the 5′ end enhances the InvS seed region’s ability to bind targets (see Fig. 2A).

This work adds to our emerging understanding of *Salmonella* sRNAs acting as molecular timers of proper virulence program activation along the pathogen’s infection cycle [18–20, 22, 23, 75]. During intracellular growth, PinT and InvS are both induced by PhoP/Q, but our *in vitro* transition assay (Fig. 2E) showed a temporal offset: PinT accumulates first, whereas InvS appears later. We do not currently know the molecular reason for this delay; it may have something to do with the genomic context and mode of biogenesis of InvS. The Hfq interface usage further supports distinct roles for the two sRNAs: PinT carries a canonical poly(U) tail and binds Hfq around nt 40–60, consistent with proximal/rim engagement, typical of class I-like sRNAs [76]. InvS harbors a U-rich 3′ tail, but contacts Hfq within an adenine-rich region (nt 60–89), suggestive of distal-face, class II-like binding [76] and in line with its greater intrinsic stability. Together, these features could yield a sequential program (Supplementary Fig. S8): early expression of PinT would clear SPI-1-associated mRNAs and delay SPI-2 induction, before InvS matures and sequesters PinT, diminishing its activity once free PinT falls below an effective threshold. As PhoP activation fades (e.g. upon bacterial escape from the intracellular environment), the stable InvS would linger in the cytosol, creating a window of opportunity for InvS to delay the production of MipA. However, future work will be required—also in regards of the relative affinities of the two sRNAs to each other, to their respective target mRNAs, and to Hfq—to test and further refine this dynamic model.

Sponge RNAs are best studied in *Enterobacteriaceae* (reviewed in [37, 38, 34]). Dating back 15 years ago, the first bacterial RNA sponge was discovered in *Salmonella enterica*: an intercistronic sequence within a chitosugar utilization operon that sequestered the ChiX sRNA [56, 77]. Five years later, bacteriophage-derived regions of the EHEC genome were found to produce stable RNAs that act as sponges of core genome-encoded sRNAs [78]. In the following, stable processing products of a precursor tRNA [79] and a polycistronic mRNA [80] were identified as sponge RNAs in *E. coli* and *Salmonella*. In the meantime, sequencing of base-pairing RNAs [40, 81] has led to the realization that RNA sponges are far more widespread and that also established mRNA-regulating sRNAs can act as decoys for other *bona fide* sRNAs, giving rise to large post-transcriptional networks [82, 34]. Independent of their origin and mode of biogenesis, sponge RNAs generally inactivate their target sRNAs through two nonmutually exclusive mechanisms: sequestering the seed region and/or promoting sRNA decay [83]. Both mechanisms are reflected in the present example: while InvS is destabilized by PinT, both sRNAs inactivate their partner sRNA by occluding its seed region. This blurs the discrimination between regulator and target for this sRNA-sRNA interaction pair.

Concomitant to the present study, PinT was reported to interact with another sRNA; the transposon-derived 5′*tnpA* [84]. As compared to PinT’s interaction site with InvS, its region base-pairing with 5′*tnpA* is further downstream. Interestingly, interaction with 5′*tnpA* did not reduce PinT’s activity (as in the case of its interaction with InvS), but seemingly enhanced PinT-mediated regulation of SPI-1 genes [84]. This highlights the intricacy of the PinT-governed post-transcriptional control network and implies that novel functional facets may await discovery even for seemingly well-characterized virulence-associated sRNAs. Molecularly understanding the complexity of these post-transcriptional virulence control circuits in bacterial pathogens bears the potential to be leveraged for anti-virulence therapeutic strategies against bacterial infections [85].

## Supplementary Material

gkaf1364_Supplemental_Files

## Data Availability

All sequencing data were uploaded on GEO under the accession number GSE293893.
