# Supplementary material for: Intramacrophage RIL-seq uncovers an RNA antagonist of the Salmonella virulence-associated small RNA PinT
Source: Nucleic Acids Res. 2025 Dec 22;53(22):gkaf1364. doi: 10.1093/nar/gkaf1364 (PMC12721325; doi:10.1093/nar/gkaf1364)
Supplement: gkaf1364_Supplemental_Files [file gkaf1364_supplemental_files.zip › Supplementary Information.pdf]

Supplementary material

Intramacrophage RIL-seq uncovers an RNA antagonist of the *Salmonella* virulence-associated small RNA PinT

Hoda Kooshapour, Gianluca Matera, Elisa Venturini, Leona Metka, Thorsten Bischler,

Jörg Vogel, Alexander J. Westermann

- Supplementary Figures S1-S8
- Legends to Supplementary Tables 1 and 2

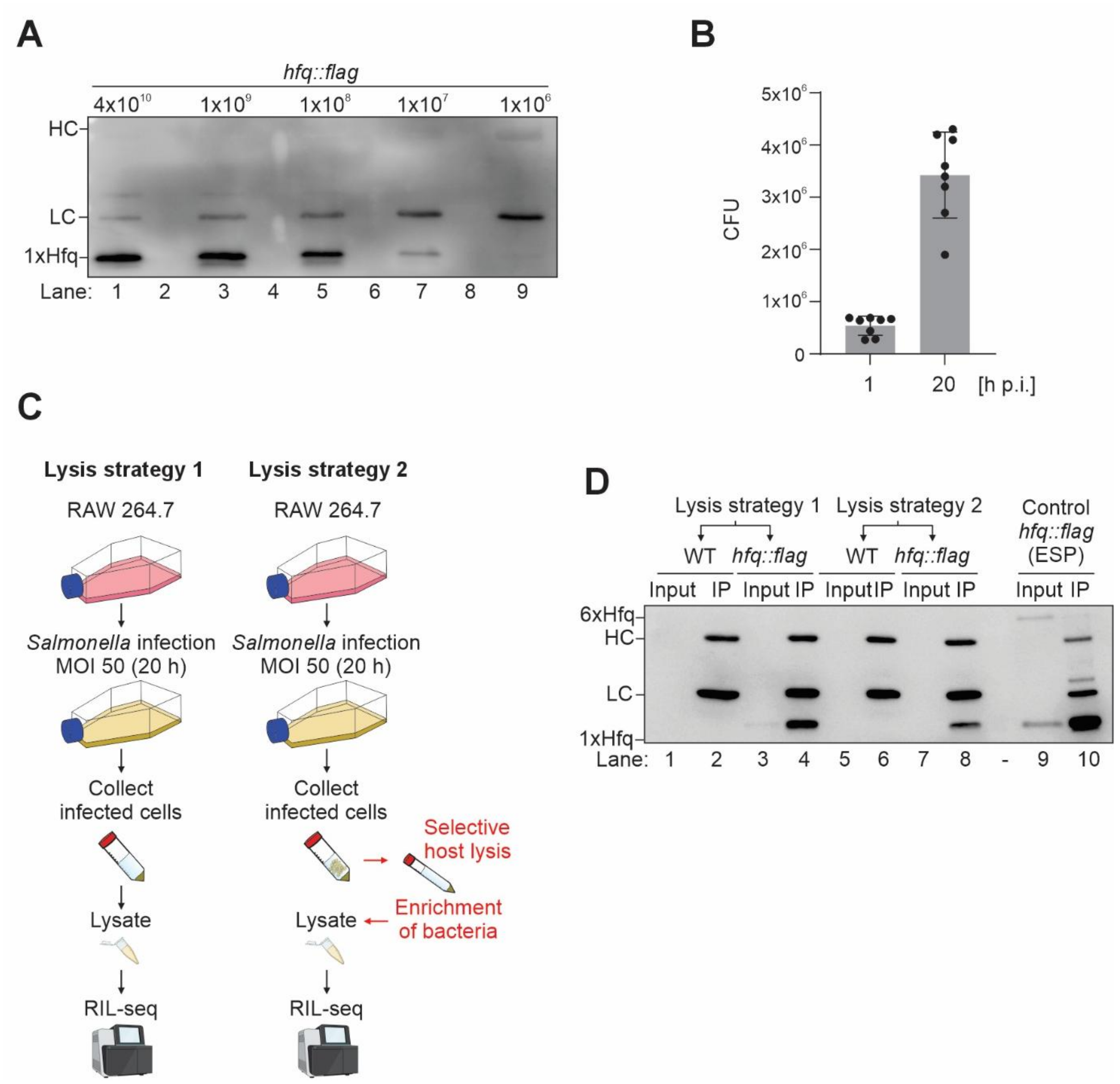

Supplementary Figure S1. Optimization of the intramacrophage RIL-seq approach. A, Western blot showing the minimum number of bacteria necessary to successfully pulldown the FLAG-tagged Hfq protein. **B,** RAW264.7 macrophages were infected at a multiplicity of infection (MOI) of 50 with wild-type *Salmonella* and the intracellular bacteria at 1 and 20 hours post infection (p.i.) were enumerated by plating assays. **C,** Two lysis strategies were evaluated for intramacrophage RIL-seq. For both of them, infected macrophages were harvested at 20 h p.i. and pelleted by centrifugation. These pellets were either directly subjected to lysis (strategy 1) or enriched for intracellular bacteria (strategy 2). In case of the latter, harvested macrophages were incubated in 0.1% PBS-Triton X-100 for 10 min at room temperature for selective lysis of only eukaryotic cells. The resulting lysate was centrifuged at 250 g for 10 min, 4°C, to pellet host cells remnants, while the released bacteria were retained in the supernatant. The supernatant was collected and centrifuged at 4,500 g for 20 min, 4°C, to also pellet the bacteria, which were then snap-frozen. **D**, Although more Hfq protein could be immunoprecipitated with the “lysis1” method, a substantially higher amount of contaminant eukaryotic RNA was present in the sample. Therefore, the percentage of reads that successfully mapped to the *Salmonella* genome was substantially lower in “lysis1” compared to “lysis2”, 10% and 34%, respectively.

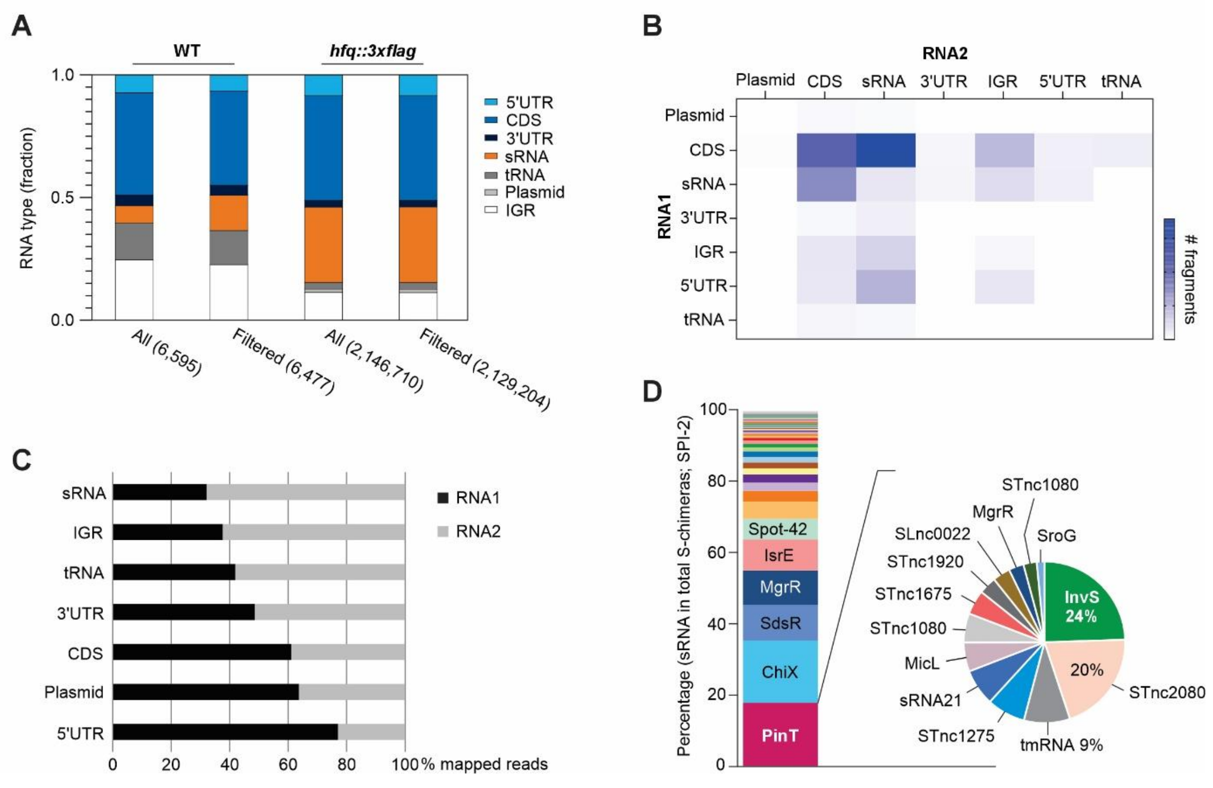

Supplementary Figure S2. Global results of the SPI-2 RIL-seq experiment. A, Relative frequency of each RNA type for significant chimeric fragments (number of fragments in S-chimeras; unfiltered [all] or filtered for ≥40 interactions) within the RIL-seq dataset derived from the wild-type and *hfq::3×FLAG* strains in the SPI-2 condition. UTR, untranslated region; CDS, coding sequence; sRNA, small RNA; tRNA, transfer RNA; plasmid, plasmid-encoded transcript; IGR, intergenic region. **B,** Heat map of the number of chimeric fragments corresponding to abundance-filtered S-chimeras according to the position of the RNA within the chimera (RNA1 = first read, RNA2 = second read). **C,** Chimeric fragments in RNA1 vs. RNA2 for each RNA type in the SPI-2 RIL-seq dataset. **D,** Distribution of all sRNAs in S-chimeras of SPI-2-inducing condition and PinT-sRNA interactome based on RIL-seq data.

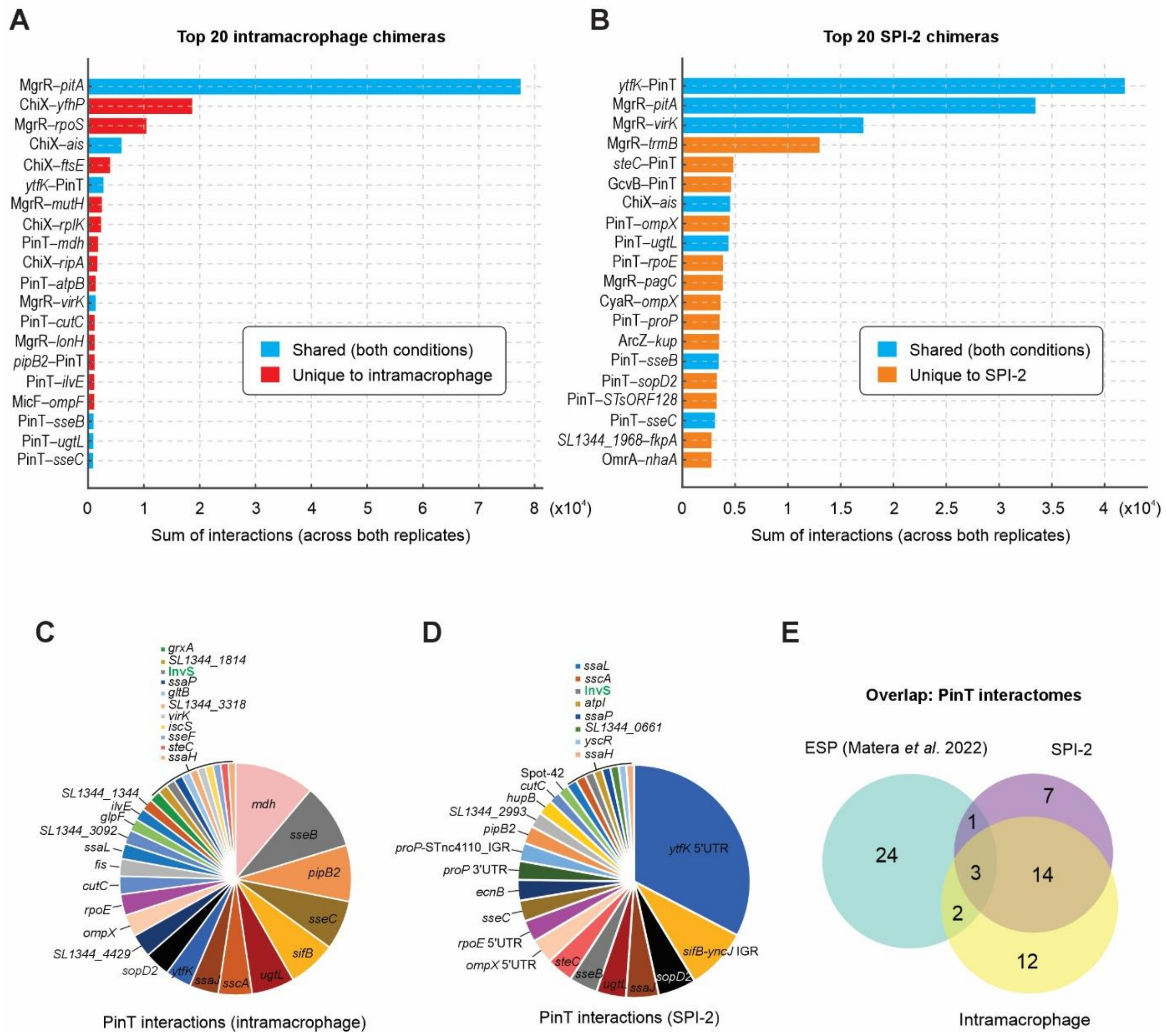

Supplementary Figure S3. Comparison of intramacrophage to conventional RIL-seq in SPI-2-inducing medium. A, B, Top 20 chimeras in intramacrophage RIL-seq (A) and in RIL-seq under the SPI-2-inducing *in-vitro* condition (B). The 2 biological replicates per each condition were summed. Shared chimeras are colored blue; significant condition-specific chimeras are in orange (SPI-2 only) or red (macrophage only). **C,** PinT interactome inside macrophages. **D,** PinT interactome under the SPI-2 inducing condition. **E,** Venn diagram illustrating the overlap of the PinT interactomes derived from 3 distinct *Salmonella* Hfq RIL-seq data sets: intramacrophage (this study), *in vitro*-growth in SPI-2-inducing medium (this study), and growth in rich medium to early stationary phase (ESP) [34]. The diagram displays the number of unique and overlapping interactions identified, highlighting shared and condition-specific interactors of PinT.

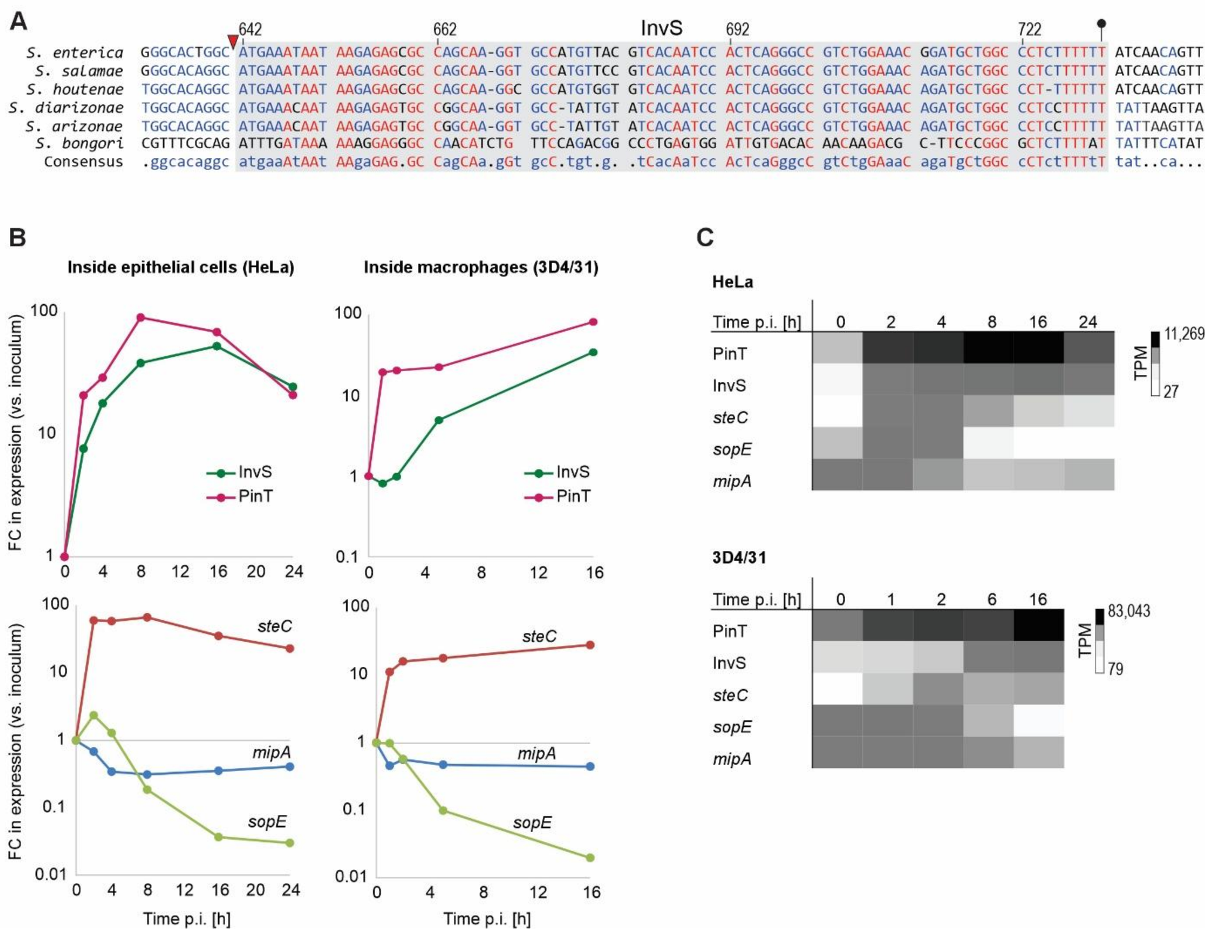

Supplementary Figure S4. Expression kinetics of InvS and PinT and of their target mRNAs by intracellular *Salmonella*. A, Sequence alignment and conservation of the *invS* locus across different *Salmonella* species. **B, C,** Relative (i.e., fold-change compared to the inoculum; panel B) and absolute (i.e., transcripts per million [TPM]; panel C) expression of PinT, InvS, and their target mRNAs during the infection of epithelial cells (HeLa) or of macrophages (3D4/31). The original RNA-seq data stem from [18].

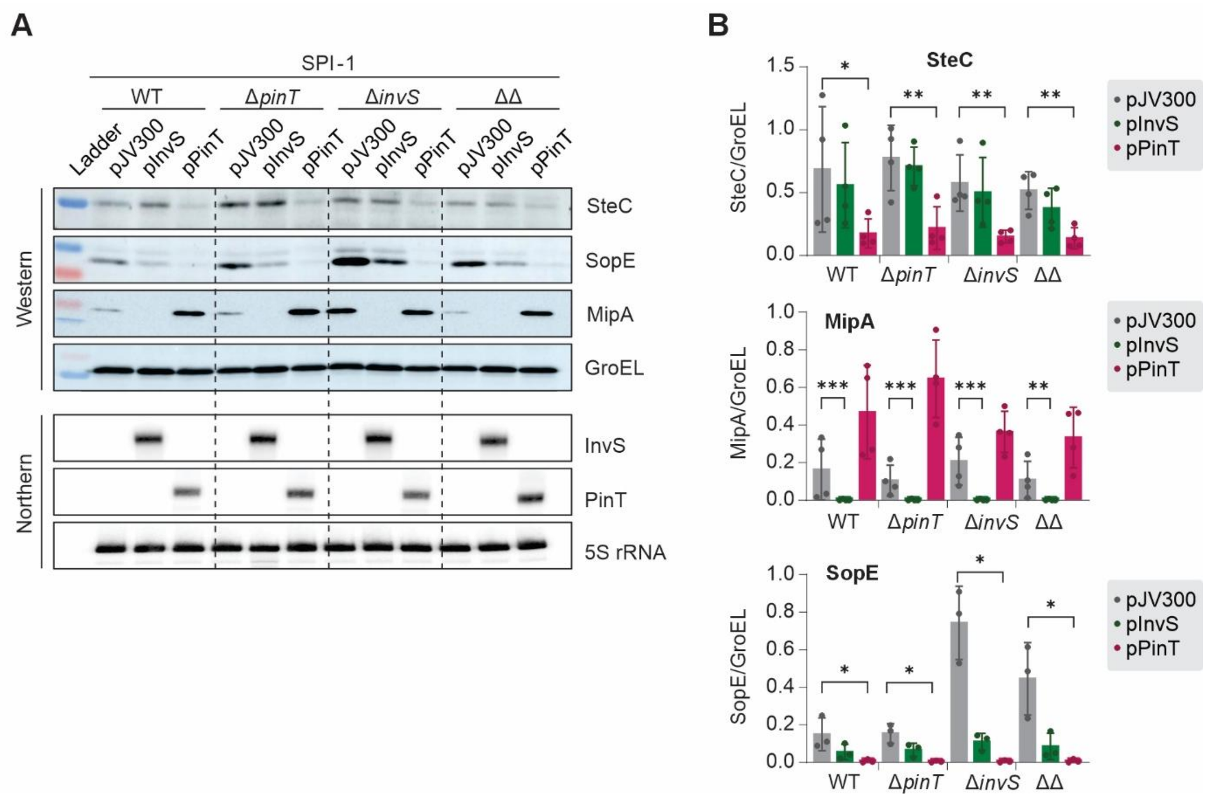

Supplementary Figure S5: Western blot under the SPI-1 condition. A, B, Western blot analysis was performed as described for Fig. 6B-C, but protein samples were collected when *Salmonella* cultures grown in LB reached an OD_{600} of 2.0 (i.e., a SPI-1-inducing condition). Panel A shows a representative result from 3 independent biological replicates and panel B the quantification over all replicates. Statistical significance was determined using lognormal ordinary one-way ANOVA. *: $p \leq 0.05$, **: $p \leq 0.01$, ***: $p \leq 0.001$.

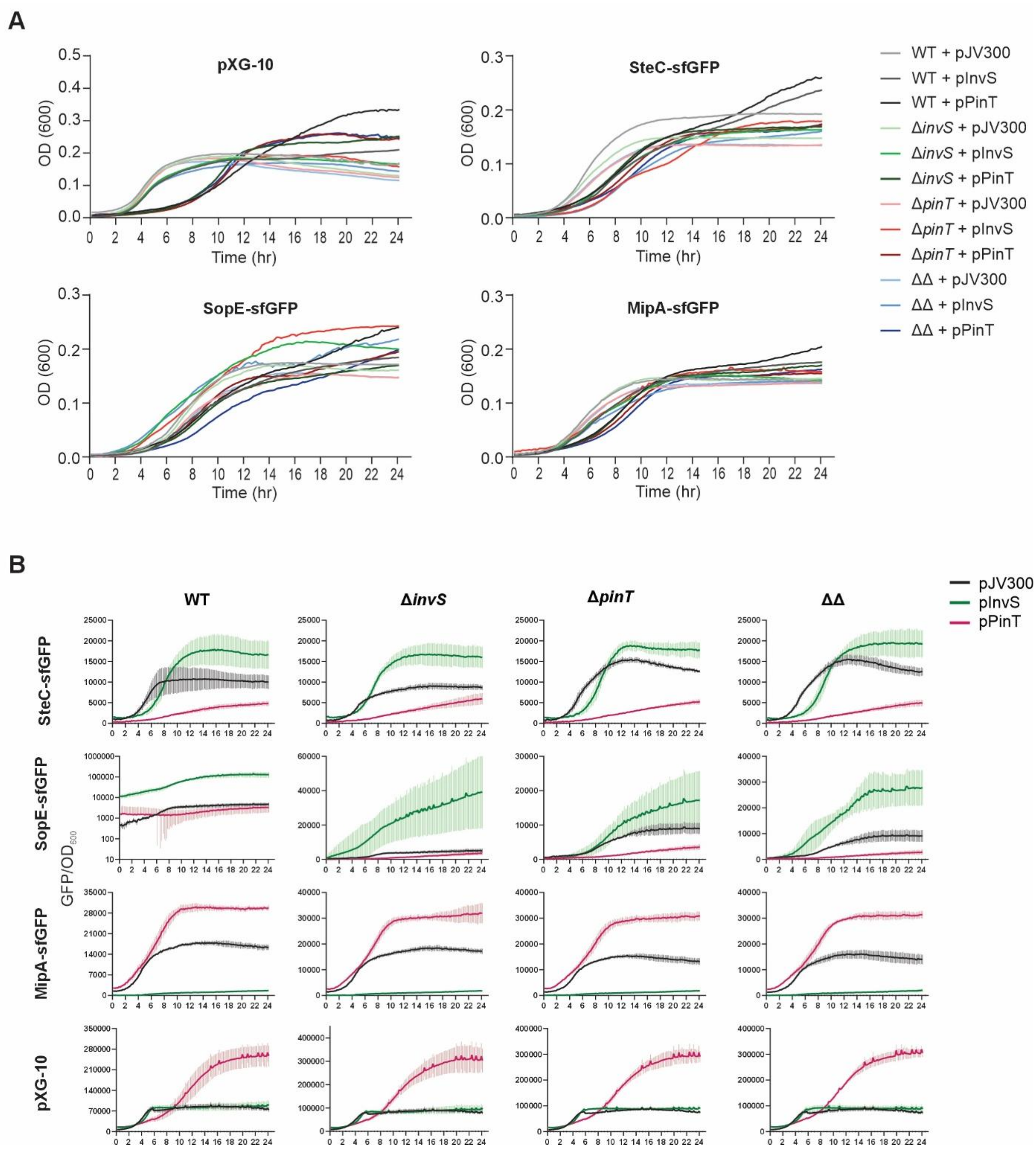

Supplementary Figure S6: Dual plasmid reporter assay of InvS and PinT targets in defined mutant backgrounds and corresponding growth kinetics. A, To monitor bacterial growth and sRNA target expression, cultures were diluted in SPI-2 MM and dispensed into a 96-well plate. Optical density was measured at 10 minute intervals throughout a 24 hour incubation. Data represent the mean of 3 biological replicates, each with 2 technical replicates. Standard deviations (SD) were omitted for clarity in visualizing the mean growth curves. **B,** A dual-plasmid reporter assay was conducted to evaluate the regulation of InvS and PinT targets in defined mutant backgrounds. Each strain was transformed with either a control plasmid or plasmids constitutively expressing InvS or PinT. Optical density at 600 nm (OD_{600}) and sfGFP fluorescence were measured at regular intervals over a 24-hour period using a plate reader. GFP expression was normalized by calculating the GFP/OD_{600} ratio for each well. Data represent the mean of 3 independent biological replicates, each measured in duplicate.

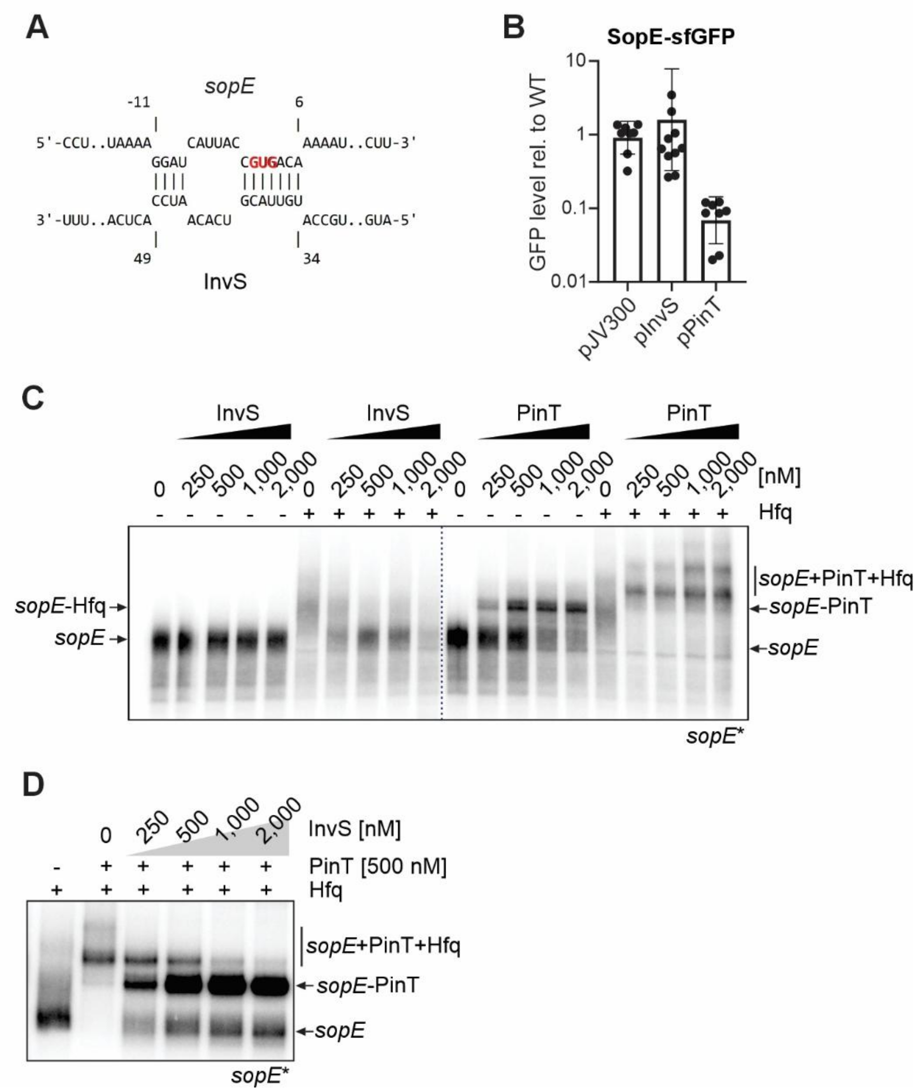

Supplementary Figure S7: Evaluation of *sopE* mRNA as an InvS target candidate. A, IntaRNA-derived prediction of InvS base-pairing to the translation initiation region of *sopE* mRNA. Numbers refer to the position relative to the 5' end of InvS or the start codon of *sopE*, which is highlighted in red. **B,** Fluorescence quantification of SopE-sfGFP translational fusion expression by flow cytometry. Strains carried either the empty vector (pJV300) or an sRNA overexpression plasmid. PinT is a known repressor of SopE [18] and was included as a positive control. Data are presented as mean ± SD from 6 independent biological replicates for pJV300- and pPinT-carrying strains, and 10 independent biological replicates for pInvS. **C,** The radioactively labeled 5′ fragment of *sopE* does not bind to InvS (left half), but binds to PinT (right half), both independently and in the presence of 100 nM of the RNA chaperone Hfq. **D,** InvS competes with PinT-*sopE* duplexes for Hfq binding. Three-component EMSA was performed by increasing concentrations of InvS in the continuous presence of Hfq (100 nM).

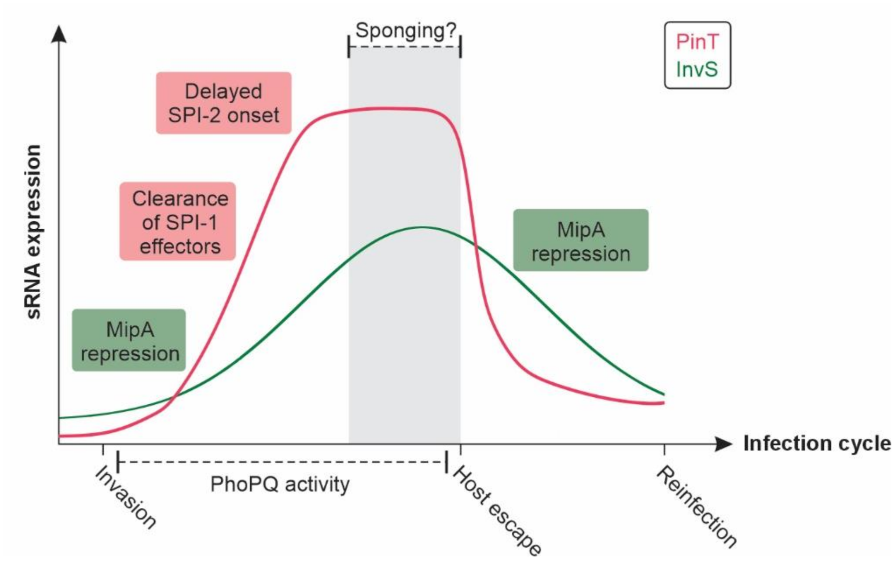

Supplementary Figure S8: Proposed dynamic model of PinT- and InvS-mediated regulations at different stages of *Salmonella* infection. While PinT and InvS are both induced by PhoP/Q, the *in-vitro* transition assay (Fig. 2E) revealed that InvS expression is delayed relative to that of PinT. Thus, shortly after host cell invasion, PinT would mediate the degradation of SPI-1-associated mRNAs and delay the onset of SPI-2 expression, before being sponged by InvS. Despite being slightly destabilized by PinT, InvS has an exceptionally long cellular half-life (>1 hr for InvS as compared to ~10 min for PinT; Fig. 5E, F). As a consequence, once the PhoP/Q activation is turned down (e.g. as *Salmonella* egresses from infected cells), InvS remains longer in the cytosol than PinT, potentially creating a window of opportunity for InvS to delay the production of the MipA adhesin prior to the reinfection of neighboring cells. We note, however, that this temporal model ignores the affinity between the sRNAs and their targets and between each other as well as to the free pool of Hfq hexamers, which—next to RNA steady-state level—represents a second factor dictating the order of events in this regulatory cascade.

Supplementary Table 1: RIL-seq data. Sheet 1: counts of sequenced and mapped fragments in individual datasets, as identified through RIL-seq. Sheet 2: significant interactions unified over the two intramacrophage RIL-seq experiments (following lysis strategy #2). Sheet 3: significant interactions unified over the two SPI-2 RIL-seq libraries. Only chimeras involving 5'UTRs, CDSs, 3'UTRs, or sRNAs were retained for further analysis. Across replicates, this number of significant chimeras was 27,150 in intramacrophage RIL-seq and 3,805 under the SPI-2 condition.

Supplementary Table 2: Bacterial strains, plasmids, oligonucleotides, and antibodies used in this study.
